# Long-range projection neurons of the mouse ventral tegmental area: a single-cell axon tracing analysis

**DOI:** 10.3389/fnana.2015.00059

**Published:** 2015-05-19

**Authors:** Ana Aransay, Claudia Rodríguez-López, María García-Amado, Francisco Clascá, Lucía Prensa

**Affiliations:** Departamento de Anatomía, Histología y Neurociencia, Facultad de Medicina, Universidad Autónoma de MadridMadrid, Spain

**Keywords:** dopamine, ventral pallidum, single-cell labeling, axonal branching, cortex, thalamus, parabraquial pigmented nucleus, rostral ventral tegmental area

## Abstract

Pathways arising from the ventral tegmental area (VTA) release dopamine and other neurotransmitters during the expectation and achievement of reward, and are regarded as central links of the brain networks that create drive, pleasure, and addiction. While the global pattern of VTA projections is well-known, the actual axonal wiring of individual VTA neurons had never been investigated. Here, we labeled and analyzed the axons of 30 VTA single neurons by means of single-cell transfection with the Sindbis-pal-eGFP vector in mice. These observations were complemented with those obtained by labeling the axons of small populations of VTA cells with iontophoretic microdeposits of biotinylated dextran amine. In the single-cell labeling experiments, each entire axonal tree was reconstructed from serial sections, the length of terminal axonal arbors was estimated by stereology, and the dopaminergic phenotype was tested by double-labeling for tyrosine hydroxylase immunofluorescence. We observed two main, markedly different VTA cell morphologies: neurons with a single main axon targeting only forebrain structures (FPN cells), and neurons with multibranched axons targeting both the forebrain and the brainstem (F + BSPN cells). Dopaminergic phenotype was observed in FPN cells. Moreover, four “subtypes” could be distinguished among the FPN cells based on their projection targets: (1) “Mesocorticolimbic” FPN projecting to both neocortex and basal forebrain; (2) “Mesocortical” FPN innervating the neocortex almost exclusively; (3) “Mesolimbic” FPN projecting to the basal forebrain, accumbens and caudateputamen; and (4) “Mesostriatal” FPN targeting only the caudateputamen. While the F + BSPN cells were scattered within VTA, the mesolimbic neurons were abundant in the paranigral nucleus. The observed diversity in wiring architectures is consistent with the notion that different VTA cell subpopulations modulate the activity of specific sets of prosencephalic and brainstem structures.

## Introduction

The projections from the ventral tegmental area (VTA) to cortical and subcortical structures occupy a central position in the neural subsystems involved in reward, prediction, and motivation as well as addiction behavior (Wise, 1978, 2002, 2009; Berridge and Robinson, 1998; Ungless, 2004; Pitchers et al., 2014; Ranaldi, 2014), and also in the physiopathology of psychiatric disorders like schizophrenia (Sesack and Carr, 2002; Laviolette, 2007; Lau et al., 2013) or major depression (Nestler and Carlezon, 2006; Friedman et al., 2009; Russo and Nestler, 2013). This ventral mesencephalic territory has been subdivided in up to eight subdivisions/nuclei whose delineation and terminology varies substantially between authors (Swanson, 1982; Ikemoto, 2007; Fu et al., 2012): parabrachial pigmented nucleus (PBP); paranigral (PN); parainterfascicular (PIF); rostral ventral tegmental area (rVTA); ventral tegmental tail (VTT); interfascicular (IF); rostral linear (RLi); and caudal linear (CLi). VTA consists mainly of dopaminergic (DAergic) neurons (65%) interspersed with GABAergic (33%) and glutamatergic neurons (2–3%) (Kawano et al., 2006; Nair-Roberts et al., 2008; Morales and Root, 2014). The largest number of DAergic neurons is concentrated in the PBP, whereas GABAergic neurons predominate in the posterior aspects of VTA (Olson and Nestler, 2007), and glutamatergic projections arise mainly from rostromedial sectors involving the RLi and the medial sectors of PBP and the rVTA (Yamaguchi et al., 2011). A growing body of data suggests that neurons in multiple brain regions may coexpress both tyrosine hydroxylase (TH), a critical enzyme for dopamine (DA) synthesis, and proteins involved in the metabolism of GABA or glutamate, suggesting that these neurons may co-release DA and amino acid neurotransmitters (Olson and Nestler, 2007). A subpopulation of neurons at the medial portion of VTA coexpresses the vesicular glutamate transporter VGluT2 and TH (Yamaguchi et al., 2011), and double-staining for these two proteins has been detected on varicosities in prefrontal cortex and the nucleus accumbens (Acb) (Yamaguchi et al., 2011; Gorelova et al., 2012). Additionally, DAergic axons were seen to release GABA using the vesicular monoamine transporter VMAT2 activity, instead of the already-known vesicular GABA transporter VGAT (Tritsch et al., 2012).

Accumulated evidence from previous studies using bulk retrograde and anterograde tracer injections indicates that VTA innervates multiple regions of the cerebral cortex and basal forebrain (Lindvall et al., 1977; Fallon and Moore, 1978; Beckstead et al., 1979; Fallon, 1981; Albanese and Bentivoglio, 1982; Porrino and Goldman-Rakic, 1982; Swanson, 1982; Oades and Halliday, 1987; Le Moal and Simon, 1991; Gaykema and Zaborszky, 1996, 1997; Carr and Sesack, 2000; Yamaguchi et al., 2011; Chandler et al., 2013; Hosp and Luft, 2013; Yetnikoff et al., 2014), but limitations of the conventional tracing methods have hampered a full understanding of the specific projection systems that originate inside VTA. For example, given the rather small size of some VTA subdivisions, conventional tracing techniques might label neurons located in several of these territories, making it impossible to ascertain whether neurons located in a given VTA subdivision have specific projection patterns or not. There are also many axons passing through VTA that may non-specifically take up tracers. Another aspect concerning the efferent projections of VTA that has not yet been analyzed are the projection systems arising from single VTA neurons through their putative axon collaterals. Knowledge about the axonal projection pattern of single neurons in the substantia nigra-VTA complex is essential for defining the neuron phenotypes and their functional role, which is fundamentally dependent on the branching pattern and terminal arbor distribution of the axons. The analysis of the axonal collateralization patterns of single nigrostriatal neurons has demonstrated the existence of very rich axonal arbors that could be confined to the striatum or also target many extrastriatal structures (Gauthier et al., 1999; Prensa and Parent, 2001; Matsuda et al., 2009; Cebrián and Prensa, 2010). In contrast, the axonal projections originating from neurons in the VTA are thought to be mainly unbranched (see Yetnikoff et al., 2014), although it is reasonable to expect that there must be several neuron phenotypes that process different aspects of reward- and motivation-related behaviors.

With the purpose of investigating the cellular diversity of the VTA projection neurons and whether the various cytoarchitectonic subdivisions of VTA innervate different or equivalent sets of targets, the present study has analyzed the efferent projections arising from the two most commonly identified VTA regions comprised of large numbers of DAergic neurons, the PBP and PN, and from a DA cell-body-poor region, namely the rVTA. This analysis has been done using two methodological approaches: (1) labeling small populations of neurons confined to a single VTA subdivision with the anterograde tracer biotin dextran amine (BDA) in order to map the general patterns of their projection systems, and (2) reconstructing the entire axonal arborization of single neurons infected with the Sindbis-pal-eGFP viral vector located in the above-mentioned VTA subdivisions. For some of the neurons whose axons were reconstructed we also provide information about their DAergic nature and the length of the terminal axonal arbor at their target structures. This study provides the first detailed information on the various projection neuron phenotypes as defined by their axonal branching patterns in the mouse VTA.

## Materials and methods

### Animals

A total of 51 adult male C57BL/6 mice, weighing between 26 and 33 g were used in the present study. All surgical and animal care procedures were carried out in accordance with European Community Council Directives (86/609/EEC and 2010/63/UE) and approved by the Bioethics Committee of our University. Animals were first anesthetized with a cocktail of 0.3 ml ketamine (Imalgène 500, 100 mg/kg) plus 0.2 ml xylazine (Rompun 2%, 4–8 mg/kg) and 0.5 ml saline administrated intraperitoneally (0.45 ml solution per 100 g body weight). Afterwards, animals were kept arreflexive under gas anesthesia (1–2% isoflurane in O_2_) through a mask attached to the stereotaxic apparatus (David Kopf Instruments, Tujunga, CA, USA).

### BDA microdeposits

In 21 mice, small deposits of the anterograde tracer biotin dextran amine (BDA 10000D, Invitrogen, California, USA) were placed bilaterally at different VTA subdivisions using the atlas of Franklin and Paxinos (2007) as reference for stereotaxic coordinates. Each microdeposit was made by delivering a small amount of 3% BDA in 0.5 M potassium acetate using glass micropipettes (outer tip diameter 3–7 μm) connected to an iontophoresis device (Units ION100-T and PS-100, Dagan Corporation), passing positive current pulses of 350–450 nA (1 s on/ 1 s off) for 30 min on average.

After a survival period of 7 days, the animals were given an overdose of anesthetic (0.2 ml of pentobarbital 16% in saline) and perfused transcardially with 15 ml of a solution containing 0.1% heparin in saline followed by 100 ml of 4% paraformaldehyde in 0.1 M phosphate buffer (PB, pH 7.4). Then the brains were postfixed in 4% paraformaldehyde for 24 h at 4°C and cryoprotected in a 30% solution of sucrose in PB for 48 h at 4°C. Brains were sagittally sectioned on a freezing microtome (Leica SM 2400) at 70 μm. To reveal the BDA-labeled axons in fine detail, all sections were processed using glucose-oxidase and an avidin-biotin-peroxidase kit (ABC; Vector, California, USA) with nickel sulfate-enhancement. The sections were next mounted on slides and stained with thionine (Panreac). Then, they were dehydrated through passage in ascending grades of alcohol, defatted in xylene for 30–40 min and coverslipped with DePeX mounting medium (Serva).

### Single cell labeling with the palGFPSindbis vector

#### Injection and fixation

We stereotaxically injected a low titration Sindbis vector solution bilaterally into VTA in 30 mice (60 hemispheres). From these experiments, we were able to recover a total of 30 individual VTA Sindbis-labeled cells valid for analysis (19 hemispheres; Table 1). The palGFP-expressing Sindbis vector diluted with 0.5% bovine serum albumin (BSA) in PBS to a final concentration of 0.5 × 10^7^ particles/ml was injected by pressure through a glass micropipette (outer tip diameter 20–40 μm) attached to a Picospritzer III (General Valve Corporation, USA). After a survival period of 48 h the mice were perfused as described above and their brains cut into 50-μm-thick sagittal sections on a freezing microtome (Leica SM 2400).

**Table 1:** **VTA neurons infected and reconstructed per animal**.

**Mice**	**Hemisphere**	**Number of infected VTA neurons**	**Number of reconstructed VTA neurons**
1	Right	2	2
2	Right	3	2
3	Left	3	1
4	Right	1	1
	Left	1	1
5	Left	1	1
6	Left	2	1
7	Right	2	1
	Left	2	1
8	Right	5	2
9	Right	15	3
10	Left	1	1
11	Right	6	3
12	Right	2	2
13	Right	4	2
	Left	3	1
14	Left	3	3
15	Left	1	1
16	Left	2	1

#### Immunofluorescence labeling for tyrosine hydroxylase (TH)

The sections including the injection site were observed under a fluorescent microscope (Eclipse E80i, Nikon) with an appropriate filter set (excitation, 450–490 nm; emission, 515–565 nm) to visualize the palGFP-expressing VTA neurons. Those sections containing palGFP-positive (+) neurons within VTA were incubated overnight at 4°C with an anti-TH monoclonal antibody (ImmunoStar; 1:1000) in PBS containing 0.1% Triton X-100 and 2% normal goat serum (NGS). All the incubations described hereafter were followed by rinses with PBS. The sections were incubated for 2 h at room temperature with an AlexaFluor 568-conjugated anti-mouse IgG goat antibody (Molecular Probes; 1:200) in PBS containing 0.1% Triton X-100 and 2% NGS. Under the fluorescence microscope with appropriate filter sets for GFP fluorescence and Alexa Fluor 568 (excitation, 579 nm; emission, > 603 nm), we determined the location of the infected neurons with respect to the various VTA subdivisions (see below in Data Analysis). Then, the palGFP-expressing-VTA neurons were examined with the confocal microscope (Espectral Leica TCS SP5; 10% argon laser and 52% DPSS 561 nm laser, 800 gain, -0.3% offset; 63× objective) to determine whether the GFP^+^ neurons also expressed TH immunoreactivity.

#### Immunoperoxidase staining for GFP and counterstaining for thionine

All the sections were then placed for 20 min at room temperature in 2% hydrogen peroxide (H_2_O_2_; 30%) to remove the endogenous peroxidase activity. After two rinses in PB with 1% Triton X-100 the sections were incubated overnight at room temperature with purified rabbit anti-GFP antibody (Exbio; 1:500), 2% Triton X-100, 3% NGS, and 1% BSA in PB. After several rinses in PB the sections were incubated for 2 h at room temperature with biotinylated goat anti-rabbit IgG (Chemicon; 1:100), 2% Triton X-100, 3% NGS and 1% BSA in PB. Finally, the sections were incubated under agitation overnight at 4°C in a solution containing ABC Elite (Vector, PK 6100) 1:100 in PB 0.1 M plus 2% Triton X-100. After several rinses in PB the bound peroxidase was revealed using the glucose oxidase-DAB-nickel method (Shu et al., 1988), and the sections were serially mounted onto gelatinized glass slides and counterstained with thionine to reveal cytoarchitecture. They were then dehydrated through passage in ascending grades of alcohol, defatted in xylene for 30–40 min and coverslipped with DePeX mounting medium.

## Data analysis

### Neurochemical delineation of the VTA subdivisions and the targeted structures

**Figure 1:**
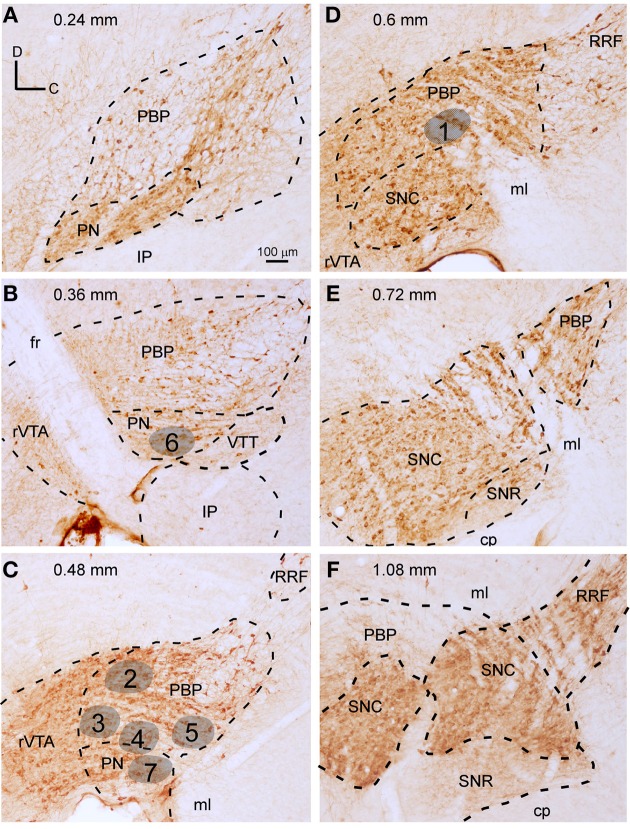
**Delineation of VTA subdivisions and location of the BDA deposits. (A–F)** Sagittal sections from the same mouse mesencephalon immunostained for TH showing the demarcation of PBP, PN, rVTA, and VTT at different mediolateral levels (values in mm at the top indicate the approximate mediolateral level). Areas in gray illustrate the location of the BDA deposits at PBP and PN, gathered in five mice (see Table 1 of Supplementary Material for further details on the BDA deposits). The scale bar in **(A)** is valid for **(B–F)**.

Sagittal sections of a mouse mesencephalon covering the complete extent of the substantia nigra pars compacta (SNC)/VTA/retrorubral field (RRF) complex were immunostained for TH to define the boundaries of the VTA subdivisions, as described by Ikemoto (2007) and the atlas of Franklin and Paxinos (2007) (Figure 1). The protocol for TH immunohistochemistry was essentially the same as that described above except that the secondary antibody was a biotinylated horse anti-mouse IgG 1:250 (Vector). Other stainings such as calbindin immunoreactivity, acetylcholinesterase (see Tripathi et al., 2010 for protocol), NADPH-diaphorase (see Scherer-Singler et al., 1983 for protocol) and Nissl were also performed in sagittal sections of the entire brains of two adult C57BL/6 mice to help in the identification and delineation of the VTA subdivisions and other brain structures.

### Localization of the BDA deposits and the palGFP-expressing neurons

**Figure 2:**
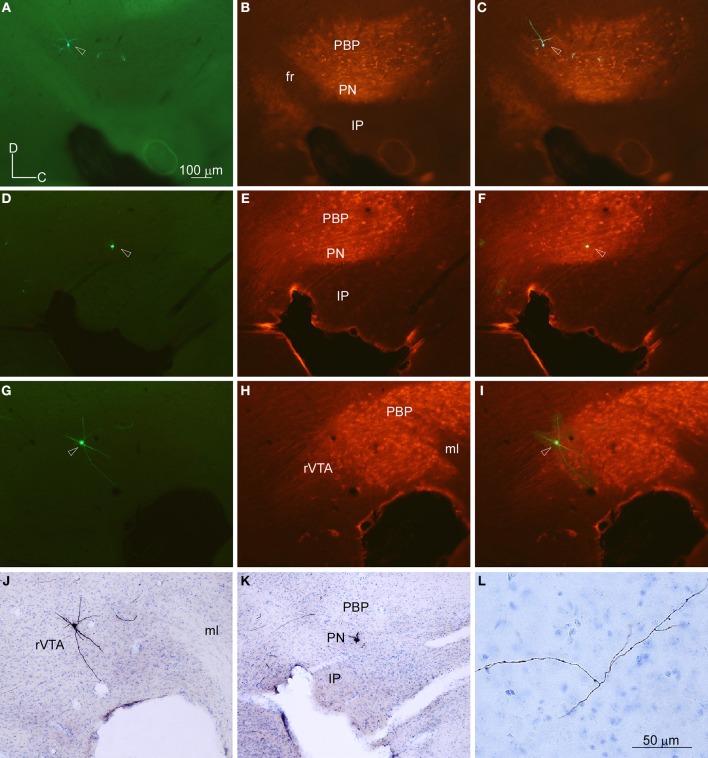
**Location of the cell bodies filled with viral vector Sindbis-pal-eGFP in the VTA subdivisions delimited by TH immunoreactivity. (A–C)** Photomicrographs taken with the fluorescence microscope from one section of the mouse mesencephalon showing one GFP-labeled neuron **(A)**, the TH-immunostaining **(B)** and the merging of both images **(C)**. Note that the neuron is at the dorsal aspect of PBP, just behind the fasciculus retroflexus (fr). **(D–F)** Photomicrographs of another sagittal section from a different mouse showing one GFP-labeled neuron **(D)**, the TH-immunostaining **(E)**, and the merging of both images **(F)**. This neuron is located at the PN. **(G–J)** Photomicrographs of another sagittal section showing one GFP-labeled neuron **(G)**, the TH-immunostaining **(H)**, the merging of both images **(I)**, and the immunoperoxidase staining for GFP and counterstaining for thionine to reveal cytoarchitecture **(J)**. Note that this neuron lies at rVTA. **(K,L)** Photomicrographs showing the cell body **(K)** and a piece of its axon **(L)** of the neuron shown in **(D)** after the immunoperoxidase staining for GFP and the counterstaining for thionine to reveal cytoarchitecture. The scale bar in **(A)** is valid for **(B-K)**.

The location of the BDA deposits was determined by cytoarchitecture. The localization of each GFP-expressing neuron was determined firstly by looking at the location of the cell body with respect to the TH-immunofluorescence labeling performed on the same section where the neuron was located (Figures 2A–I). Secondly, after revealing the GFP and the counterstaining with thionine of that same section containing the cell body, this location was corroborated by cytoarchitecture (Figures 2J–L). Since this study was aimed at analyzing the efferent projections of single VTA subdivisions, only those BDA deposits that were confined to one subdivision were included in the study (see Table 1 in Supplementary Material for details of the BDA deposits analyzed). Tissue sections containing the BDA deposits and their adjacent sections were thoroughly examined to detect putative retrogradely labeled cells and only neurons located at the surroundings of the tracer deposits were found. Most of these neurons were very close to the BDA deposit, being located at the same VTA subdivision, and were intensely labeled, with clearly impregnated cell body and dendrites. However, those neurons located further from the BDA deposit showed a very weak labeling of their cell body and dendrites, probably due to insufficient tracer uptake due to the very small amount of BDA delivered in each deposit and, therefore, their axons were considered unlikely to provide any visible terminal field.

The terminal fields were examined under a microscope with 20 and 40× objectives for detailed analysis and photomicrographs were taken using a digital camera (DXM 1200F, Nikon) attached to the microscope (Eclipse E80i, Nikon). The final figures were adjusted in brightness and contrast and sharpened using Adobe Photoshop CS4 software (v. 11.0, Adobe). Photomicrographs in **Figures 5B**, **7C** are merged images of Z stacks of photographs taken at different planes of focus using the Image J software (Rasband, 1997–2012).

### Single neuron tracing

The injections of the Sindbis vector resulted in a total of 15 out of 19 injected hemispheres that had only 1–3 infected neurons (Table 1). Axons in these experiments could be readily individualized and traced across the serial sections. The remaining four hemispheres contained 4, 5, 6, and 15 labeled cells, and we were able to separate and reconstruct confidently 10 additional cells from these cases. Those neurons whose axons were entangled or overlapped the axons of other neurons and were inseparable for reconstruction were left out of this study.

The cell bodies and entire axonal trajectories of palGFP-expressing VTA neurons were drawn and registered to tissue landmarks from the serial sagittal sections using a microscope (Nikon Eclipse 50i) with 20 and 40× objectives and a camera lucida. Drawings were subsequently digitalized with a scanner, and the axons were redrawn using CANVAS X software (ACD Systems International).

### Stereological estimations

Since DA release from VTA neurons might occur at synaptic and extrasynaptic sites, knowing the length of the terminal axonal arbors could provide a clue about the strength of VTA innervation in the targeted structures. To this end, we estimated the length of the terminal axonal arbor present at the main structures innervated from single mesocorticolimbic, mesolimbic, and mesostriatal Sindbis-infected VTA neurons to allow comparison of the length of terminal arbors provided by different VTA neuron types. Additionally, we have obtained the length of the total terminal axonal arbor provided by each of these neuron types. These measurements could not be made in the two mesocortical VTA neurons since their axons merged at the cerebral cortex, making it impossible to separate the terminal arbors provided by each neuron. In the case of the forebrain- and brainstem-projecting neuron type (F + BSPN), measurements were not made due to the scarce innervation that these neurons provided at their target structures.

We used the isotropic virtual planes method (Larsen et al., 1998) to estimate the length of terminal axonal arbors; methods have been described in detail elsewhere (García-Amado and Prensa, 2013). For that purpose, a BX61 microscope (Olympus) equipped with a microcator (0.5 μm resolution), a motorized ProScan II stage (Prior Scientific), and a digital DP-71 camera (Olympus) connected to a computer with two screens was used. The newCAST stereology software package from VIS (Visiopharm Integrator System; Hørsholm, Denmark; v. 3.6.2.0) was employed.

Measurements of the length of terminal axonal arbors started at the point where the main axon or its branches entered the boundaries of the targeted structure. Depending on the size of the terminal arbors, we selected some sections with a random start separated by a constant interval, obtaining around 7–9 sections per case. Each terminal arbor was delineated at 10× magnification and then superimposed with a grid of sampling boxes containing the virtual planes generated by the software. The intersections between the fibers and the planes were counted. We kept an upper and lower guard zone along the z axis of the sections where fibers were not counted. The upper guard zone was always 3 μm and the size of the lower guard zone resulted from subtracting the upper guard zone plus the height of the sampling box from the section thickness (see Table 2 of the Supplementary Material for further details). The parameters of the grid and the sampling box were chosen in order to obtain approximately 100–200 intersections per terminal arbor.

## Results

### Delineation of the PBP, PN, and rVTA subdivisions in mice

An unambiguous delineation of the various VTA subdivisions is important to analyze and compare the efferent projection systems that arise from each subdivision. In the present study, the VTA subdivisions PBP, PN, and rVTA were outlined in sagittal sections of one mouse mesencephalon stained for TH, which identifies solely DAergic neurons in these regions (Fu et al., 2012), based on the atlas of Franklin and Paxinos (2007) and the delineation proposed by Ikemoto (2007). PBP and PN are DAergic (TH^+^) cell-body-rich zones, whereas the rVTA contains notably fewer DAergic cell-bodies (Figure 1). A similar scarceness of TH^+^ neurons occurs at the posterior aspect of VTA, which corresponds to the VTT subdivision delimited by Ikemoto (2007), posterior to PN, slightly ventral to the caudal pole of PBP and above the IP (Figure 1B).

The largest VTA subdivision is PBP, stretching from 0.24 to 1.08 mm lateral to the midline (Figure 1). PBP lies dorsal to PN and posterior to the fasciculus retroflexus (fr) and rVTA from 0.24 to 0.48 mm (Figures 1A–C); the PBP has a lower TH^+^ neuropil density and slightly larger TH^+^ cell bodies than PN. At 0.6 mm, PBP lies above SNC, rostral to RRF, and caudal to rVTA (Figure 1D). PBP and SNC contain densely packed TH^+^ cell bodies, and the boundaries between these structures are vaguely defined. At level 0.72 mm the medial lemniscus (ml) separates PBP from SNC (Figure 1E), and from 0.84 mm to 1.08, PBP lies between the ml and SNC (Figure 1F).

The PN occupies a notably smaller volume than PBP, stretching from 0.24 to 0.48 mm lateral to the midline (Figures 1A–C). Medially it lies between PBP and the interpeduncular nucleus (IP) (Figures 1A,B) and shows dark TH^+^ staining, its DA cells being smaller and more tightly arranged than those at PBP, with their somatodendritic domain oriented along the horizontal plane.

The rVTA occupies the rostral-most territory of the VTA from level 0.36 to 0.6 mm lateral to midline (Figures 1B–D), lying rostral to the fr, PBP, PN, and SNC. Compared to PBP and PN, the rVTA has a lower density of TH^+^ cell bodies, which are smaller and more weakly stained in the medial aspect of this zone than laterally.

### Terminal axonal fields labeled from BDA microdeposits at PBP and PN

**Figure 3:**
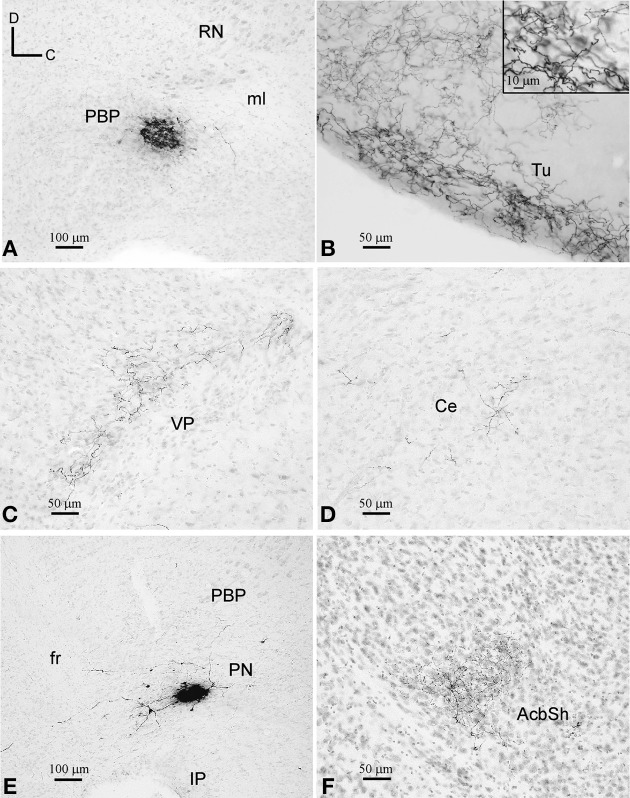
**BDA deposits and labeled terminal fields. (A–D)** BDA deposit at PBP **(A)** and terminal fields at the Tu, VP, and Ce **(B–D)**, as seen in sagittal sections stained with thionine. Note the different fiber density depending on the target structure. **(E,F)** BDA deposit at PN and terminal fibers at the AcbSh, as seen in sagittal sections stained with thionine.

To provide a population level view of the outputs of the VTA subdivisions we placed BDA microdeposits at different mediolateral and rostrocaudal levels. Here we show seven BDA microdeposits that were exclusively confined within a single VTA subdivision, five placed in PBP and two in PN (Figure 1). None of the BDA deposits at rVTA were confined to that subdivision, but they invaded either the fasciculus retroflexus or the IP, and therefore were discarded from the study. The BDA microinjection produced a dense small volume of heavily-stained neuropil and somata surrounded by two to ten heavily-stained cell bodies (Figures 3A,E). Most of these BDA-impregnated neurons were in the proximity of the BDA deposit and, therefore, confined to the same VTA subdivision. When other rare BDA-labeled neurons were observed at a distance from the BDA deposit, their labeling was extremely weak, with hardly visible dendrites and, therefore, their axons were not considered to provide any labeled terminal field. Since no other cell bodies were labeled outside this area, we assume that all labeled axons originate in the cells contained in the BDA deposits.

#### BDA microdeposits at PBP

One of the BDA microdeposits was located at 0.6 mm from midline and extended medially to 0.48 mm and laterally to 0.72 mm, spreading very slightly to the SNC. The other four BDA deposits were placed more medially, approximately at 0.48 mm, and extended up to 0.36 mm (Figures 1, 3A). One of these deposits very slightly invaded the PN but, since the distribution of the axonal terminal fields was similar to that of the other deposits located exclusively within PBP, we did not discard it. Because the animals received bilateral injections into PBP the putative contralateral projections could not be assessed in this analysis (see Table 1 in Supplementary Material).

##### Distribution of the terminal fields and intensity of innervation

The terminal axonal fields were composed of thin collaterals bearing varicosities or clusters of axon terminals. A single BDA deposit resulted in terminal axonal fields of different sizes and fiber density depending on the target structure (Figures 3B–D). For this reason, based on visual observation, we have qualitatively distinguished high, medium and low innervations in the various target structures of each BDA deposit, as shown in Table 2.

**Table 2:** **Brain structures innervated by each PBP deposit and intensity of innervation**.

**BDA deposit**	**Cerebral cortex**	**Olfactory structures**	**Striatum and ventral pallidum**	**Septal nuclei and DBB**	**Amygdaloid complex**	**Thalamus and epithalamus**	**Hypothalamus and PAG**	**SNC, VTA, RRF**	**Other brainstem regions**
1	LO	AOM,AOP	AcbSh	LSD	BL	AM	LH	PBP	PnO
	Pir	AOV	CPu	LSI	CeC,CeL	LHb	MCLH	SNC	DpMe
	Cg1,Cg2	ICj	AcbC		CeM		PH	RRF	LDTg
	DEn,VEn	Tu	VP		BSTLD,BSTLP		AHP		
	FrA	VTt					LPO		
	PrL	DTT					PAG		
	AID,AIV								
	DP								
	GI								
	S1								
2	Cg1,Cg2	AOM	AcbSh	VDB		CM	MM		PnO
	FrA	Tu	VP	LSI		MDM	PH		LDTg
	PrL	VTt		MS		PVP	SuMM		
	DP	DTT				Rh	PAG		
	GI								
	RSG								
3	Cg1,Cg2	AOM	AcbSh	LSI	BL		LH		
	DEn,VEn	DTT	CPu	MS	CeC		SuMM		
	FrA		VP		MeAD				
	MO								
	PrL								
	M2								
	VO								
4	Cg1,Cg2	AOP,AOV	AcbSh		BSTLD,BSTLP		PAG		
	DEn	Tu	AcbC						
	PrL								
	AID,AIV								
	DP								
	M1,M2								
	RSG								
	RSA								
	VO								
5	FrA						LH	RRF	LDTg
	PrL						PAG		
	DP								
	MEnt								
	RSG								
	RSA								

*Intensity of innervation: 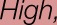

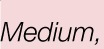
 Low. See list of abbreviations*.

The most intensely innervated regions were related to the olfactory system, such as the anterior olfactory area (AO), island of Calleja (ICj), Tu (Figure 3B), ventral tenia tecta (VTt) and the piriform cortex (Pir). PBP also projected to multiple other cortical areas, either with high intensity in the case of the lateral orbital cortex (LO), which is a major source of inputs to DAergic VTA neurons (Watabe-Uchida et al., 2012), or more moderately in the cingular (Cg), frontal association (FrA), medial orbital (MO), prelimbic (PrL), as well as the endopiriform claustrum (DEn, VEn). A few cortical terminals were also observed in the agranular (AI) and granular insular (GI), dorsal peduncular (DP), primary (M1) and secondary (M2) motor, medial entorhinal (MEnt), retrosplenial granular (RSG) and agranular (RSA), primary somatosensory (S1) and ventral orbital (VO) cortices.

The CPu and the accumbens *shell* (AcbSh) were intensely innervated, whereas the AcbC, VP (Figure 3C) and LS received moderate innervation. The amygdaloid complex [basolateral (BL), central (Ce), and medial (Me) nuclei], medial septal (MS), and lateral habenular (LHb) nuclei were sparsely targeted (Figure 3D). Within the diencephalon and brainstem, the more densely innervated structures were the lateral hypothalamic area (LH), the magnocellular nucleus of the lateral hypothalamus (MCLH), and the oral part of the pontine reticular nucleus (PnO). Other diencephalic and brainstem structures shared light labeling with low intensity (Table 2).

#### BDA microdeposits at PN

The two BDA microdeposits in PN were located at 0.36 and 0.48 mm lateral from midline (Figures 1, 3E).

##### Distribution of the terminal fields and intensity of innervation

The neural structures innervated by each BDA microdeposit and the degree of innervation observed in each of the targets, estimated qualitatively, are summarized in Table 3. The morphological features of the terminal axonal fields labeled from PN neurons were very similar to those observed with PBP neurons and mostly composed of thin varicose axons.

**Table 3:** **Brain structures innervated by each PN deposit and intensity of innervation**.

**BDA deposit**	**Cerebral cortex**	**Striatum and ventral pallidum**	**DBB**	**Amygdaloid complex and hippocampus**	**Thalamus and epithalamus**	**Hypothalamus and PAG**	**SNC, VTA, RRF**	**Other brainstem regions**
6	MEnt		HDB	Subiculum	Rt	LPAG		DTgP
	LEnt			ACo	LHbM			DpG
				BMP				LDTg
				PMCo				Pn
								APT
								MPT
7	Pir	AcbSh	HDB	ACo	AM	AHP,AHC	rVTA	DRD,DRV
		Tu			CM	LH		DTgP
		VP			MDM	LPAG,VLPAG		LDTg
					PVP			Pn
					Re			
					LHbM			
					MHb			

*Intensity of innervation: 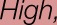

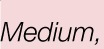
 Low. See list of abbreviations*.

As observed in PBP, the PN neurons labeled from the BDA deposits also project with different intensities to multiple cortical and subcortical structures, but the projections to the cerebral cortex from PN were more markedly restricted than those of PBP neurons. The structures most intensely innervated from PN were the MEnt and LEnt cortices, the AcbSh and Tu (Figure 3F). Terminal axonal fields of moderate intensity abounded at the Pir and secondary somatosensory (S2) cortices, VP, subiculum, anterior hypothalamic area, LH, lateral (LPAG), and ventrolateral (VLPAG) periaqueductal gray matter and certain thalamic and brainstem nuclei specified in Table 3. The cortical (Co) and basomedial (BM) amygdaloid nuclei, as well as the LHb and medial habenular (MHb) nuclei were sparsely innervated.

The results gathered from the analysis of the BDA microdeposits placed in PBP and PN indicate that these subdivisions possess neurons with widespread cortical and subcortical prosencephalic and brainstem projections. Since the BDA microdeposits invariably label small groups of neurons, these findings suggest that, at least within these two main VTA subdivisions, there are either distinct types of closely intermingled projection neurons or single neurons whose axons are extensively ramified to innervate widely scattered brain regions. To ascertain whether the patterns of labeling observed with the BDA microdeposits reflect the axon morphology of individual cells or a mixture of different cell phenotypes, we undertook the task of labeling single neurons located in different VTA subdivisions and tracing their entire axonal arborization.

### Axonal branching pattern of single neurons located in PBP, PN, and rVTA

A total of 30 neurons infected with the GFP-producing Sindbis viral vector have been analyzed here. These neurons were located in PBP (17 neurons), PN (5 neurons) and rVTA (7 neurons). The last neuron was located in the VTT region delimited in the present study, which corresponds to the TH- poor zone located just posterior to the PN (see Ikemoto, 2007) and does not represent what is currently identified as the tVTA/RMTg (Barrot et al., 2012; Yetnikoff et al., 2014). For each neuron we first examined whether it expressed TH (which is a reliable marker for the DAergic neurons in the ventral mesencephalon (Fu et al., 2012), and then we entirely reconstructed its axon. Of the 30 neurons analyzed here, five located in the PBP, PN and rVTA were shown to be DAergic. Although the other 25 neurons were not demonstrated to express TH, we cannot be sure that at least some of them did not use DA as a neurotransmitter (see Methodological Considerations in the Discussion).

We observed two markedly different VTA projection neuron phenotypes: (1) forebrain-projecting neurons whose axons innervated only forebrain structures, and (2) forebrain- and brainstem-projecting neurons (F + BSPN) with axons that branched abundantly in the proximity of the cell body and projected to numerous and widespread brain structures.

#### Forebrain-projecting VTA neurons

A total of eighteen cells (60% in our sample) had a single main axonal trunk extending rostrally to innervate only cortical and/or subcortical forebrain structures. However, since these neurons showed a striking diversity and specificity in the sets of structures targeted, we tentatively subdivided them into four different “subtypes”: (1) “Mesocorticolimbic” neurons projecting to both the neocortex and basal forebrain; (2) “Mesocortical” neurons projecting almost exclusively to the neocortex; (3) “Mesolimbic” neurons innervating the basal forebrain, AcbC and CPu; and (4) “mesostriatal” neurons targeting exclusively the CPu.

##### Mesocorticolimbic neurons

**Figure 4:**
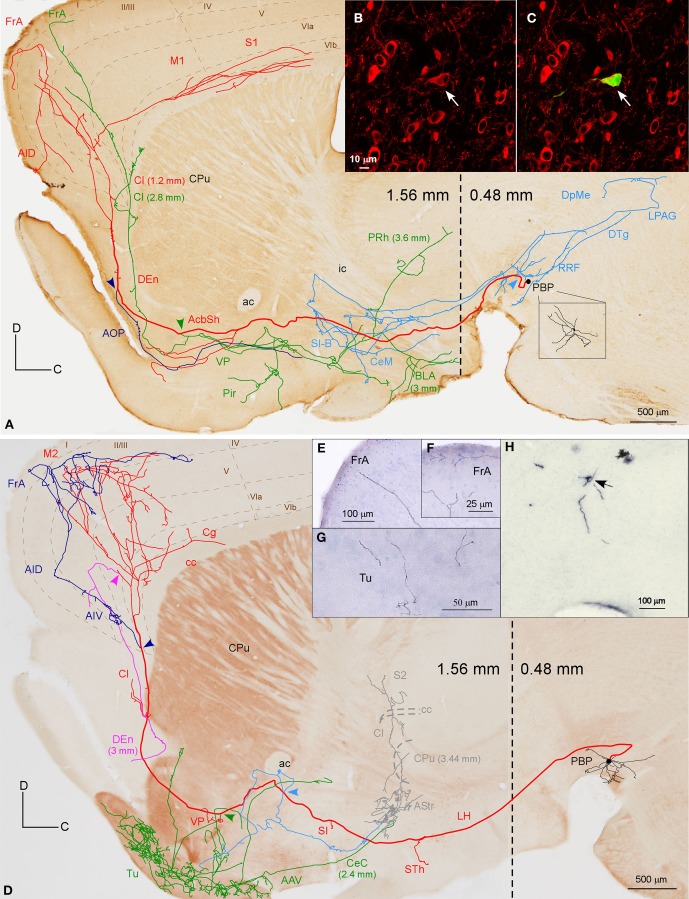
**Mesocorticolimbic neurons. (A)** Sagittal reconstruction of a single PBP mesocorticolimbic DAergic neuron superimposed over a calbindin (CB)-stained composition of two different mediolateral levels (0.48 and 1.56 separated by a dashed line) according to the atlas of Franklin and Paxinos (2007). The main axon (thick and red) reaches the cerebral cortex after providing three main collaterals that are indicated from caudal to rostral with light blue, green and dark blue arrowheads. Terminal axonal fields placed at mediolateral levels that do not correspond to the level of the CB-section are indicated between parentheses. **(B,C)** Confocal microscopy images showing the soma filled with viral vector Sindbis-pal-eGFP (green) and its TH immunoreactivity (red). **(D)** Sagittal drawing of a PBP mesocorticolimbic neuron superimposed over an acetylcholinesterase (AChE)-stained composition in which the dashed line separates a medial section containing PBP (0.48 mm) from a more lateral one (1.56 mm). The main axon (thick and red) reached the cerebral cortex after providing three main collaterals indicated with light blue, green, and dark blue arrowheads. Terminal axonal fields provided by the light blue and green collaterals that reach the AStr, CPu, Cl, and S2 at very lateral levels are indicated in gray color. **(E–H)** Photomicrographs showing terminal fibers at the cerebral cortex **(E,F)** and Tu **(G)**, and the neuron cell body at PBP **(H)**.

Two neurons out of 30 (6.6%) were observed to innervate the cerebral cortex and limbic structures of the basal forebrain including the Tu, VP, and amygdaloid complex (Figure 4, Table 4). The two somata were found in the medial aspect of PBP (0.48 mm lateral to midline) and the trajectory and collaterals of their axons are depicted in Figures 4A,D. One of these neurons was demonstrably DAergic (Figures 4B,C; see also **Figures 8D,G**). The two neurons had round-shaped perikarya from which emerged 4 to 6 poorly ramified dendrites.

**Table 4:** **VTA neuron projection phenotypes**.

**Neuron phenotype**		**Location (mediolateral level; Figures)**	**Neurotransmitter**	**Target structures**
**Forebrain-projecting neuron types**	Mesocorticolimbic	PBP (0.48 mm; Figure 4A)	DA	FrA, AID, M1, S1, PRh, DEn, Cl, AOP, SI, B, BLA, CeM, MeAD, LPAG, PBP, RRF, DTg, DpMe
		PBP (0.48 mm; Figure 4D)	−	FrA, AID/AIV, **M2**, S2, DEn, Cg, Cl, **Tu**, CPu, STh (anteromedial), SI, VP, **AStr**, AAV
	Mesocortical	PBP (0.6 mm; **Figure 5A**)	−	**S1**, Cl, LGP, SI, B, PBP (contralateral), RRF
		rVTA (0.48 mm; **Figure 5A**)	−	**S1**, S2, LGP, SI, B
	Mesolimbic	PBP (0.6 mm)	−	**AcbC**, LSS
		PBP (0.6 mm)	−	**AcbC**
		PBP (0.48 mm; **Figure 5C**)	DA	**Tu**, VP, AAV
		PBP (0.36 mm)	−	**AcbC**, CPu, SI, BSTLP (all targets were contralateral)
		PBP (0.36 mm)	−	CPu (ventrolateral), IPAC, CeC, B
		PN (0.36 mm)	−	**Tu**, Pir, VP, AAV, ACo, PBP, PN, CLi
		PN (0.36 mm)	−	Tu, AOM, BSTMPL, LPO
		PN (0.24 mm; **Figure 6A**)	DA	AcbC, BSTL, LS, LH
		PN (0.24 mm)	−	**LSV**
		PN (0.24 mm)	DA	**Tu**, **ICj**, VP, LH
		rVTA (0.6 mm)	−	**AcbSh (lateral)**, CPu (ventral)
		rVTA (0.6 mm)	DA	AcbSh (lateral), CPu (dorsocaudal)
	Mesostriatal	PBP (0.6 mm; **Figure 7A**)	−	**CPu**, LSS
		rVTA (0.6 mm)		**CPu** (dorsal), LSS
Forebrain- and brainstem-projecting neuron type (F + BSPN)		PBP (0.72 mm)	−	MDL, LH (ipsilateral), LPAG, PVG, RRF, PBP, SNC, DpMe (ipsilateral), Dk, RI, **IO** (bilateral) (rest of targets were contralateral)
		PBP (0.72 mm; **Figure 7D**)	−	PVG, PCom, DpMe, LPAG
		PBP (0.6 mm)	−	HDB, BSTMA, **LH**, LPO, MPA, ZI, PBP, rVTA, IF, PN (contralateral), IP (contralateral), PTg, DTg, **PMnR**, PnV
		PBP (0.6 mm)	−	SI, **BM**, BSTLI, LH, RM, PBP, IF, DpMe
		PBP (0.48 mm)		PSTh, SI, LPAG, PBP, RRF
		PBP (0.36 mm)	−	SI, B, Me, CeM, BSTMPL, Rt, **LH**, ML, LPO, LDTg
		PBP (0.36 mm)	−	**PH**, LPAG, PBP, rVTA, SNC, LDTg
		PBP (0.36 mm)	−	**HDB**, SI, BSTMPI, PH, ZI, PVG, PBP, LDTg, RtTg
		rVTA (0.6 mm)	−	**E/OV**, **LS**, AcbC, BSTMPL, PV, Re, Rh, Sub, MHb, AH, LH, DR
		rVTA (0.6 mm)	−	HDB, VDB, LSI, PVA, Rt, MCPO, PH, ZI, **LDTg**
		rVTA (0.36 mm)	−	HDB, RM, DMPAG
		VTT^*^ (0.36 mm)	−	**LSI**, PV, LPO, LH, **LPAG** (bilateral), PPT, OT, RtTg

Nuclei in bold case indicate the structure/s in which the axon of each neuron provided a denser collateralization than in its remaining targets, as estimated by visual observation. See list of abbreviations.

* *This neuron was located in the TH- poor zone located just posterior to the PN, named as VTT by Ikemoto (2007). This region does not represent what currently is identified as the tVTA/RMTg (Barrot et al., 2012; Yetnikoff et al., 2014)*.

The axons of both mesocorticolimbic neurons had the following common features: the main axonal branch traversed LH and the substantia innominata (SI) emitting some thin and short collaterals at these areas, and, as it traversed the accumbens (Acb), it gave off collaterals toward the basal forebrain. Finally, it innervated the claustrum (Cl) before targeting the AI and FrA (Figures 4A,D).

The specific features of these two axons can be observed in Figure 4. The axon of the DAergic neuron (Figures 4A–C) emitted, within the PBP, one collateral (light blue color) that innervated the RRF, the deep mesencephalic nucleus (DpMe), the LPAG, the dorsal tegmental nucleus (DTg) and the PBP itself, before reaching the SI-basal nucleus of Meynert (SI-B) and the medial division of the Ce (CeM). A second collateral (green color) was emitted as the main axon traversed the AcbSh and this collateral targeted VP, Pir and more laterally BL and the perirhinal cortex (PRh). It also innervated the Cl and the FrA. As the main axon traversed the Cl it provided abundant varicosities or *boutons en passant*, and a third collateral (dark blue color) that headed ventrally through the posterior part of the anterior olfactory area (AOP) toward VP. Finally, the axon innervated the FrA, the dorsal agranular insular cortex (AID), and the deep layers of the M1 and S1 cortices.

The axon of the second mesocorticolimbic neuron (Figures 4D–H) provided a short collateral at the subthalamic nucleus (STh) and, before entering Acb, it gave off its first long collateral (light blue), which ran laterally to profusely innervate the AStr before targeting the most lateral aspect of the CPu, the Cl, and S2. As the main axon traversed the VP it emitted a second collateral (green) that extensively targeted the Tu and the anterior amygdaloid area (AAV), then continued toward the capsular part of the Ce (CeC) and the AStr where it joined the first collateral and innervated the same lateral aspect of CPu, Cl, and S2. As the main axon approached the cortex it emitted some thin branches at Cl as well as a third collateral (dark blue) that innervated the AI and FrA. Finally, it branched into numerous varicose fibers that spread in all layers of the M2 as well as the deep layers of the S2 and Cg, giving a last fiber that headed ventrally and laterally toward the DEn. The length of the terminal axonal arbor that this neuron provided in M2/FrA, Tu and the AStr was of 68.31, 31.92, and 11.99 mm, respectively (Table 5).

**Table 5:** **Length of terminal axonal arbors labeled from single VTA neurons**.

**Neuron type (VTA subdivision; Figures in which it is illustrated)**	**Innervated structure**	**Length of terminal arbor (mm)**	**CE**
**Mesocorticolimbic** (medial PBP; Figure 4D)	M2/FrA/AIV	68.31	0.102
	Tu	31.92	0.103
	AStr	11.99	0.121
	S2	1.28	0.270
Total length	-	113.5	0.165
**Mesolimbic** (medial PBP; Figure 5C)	Tu	86.82	0.095
	VP	6.14	0.094
	AAV	7.54	0.131
Total length	-	100.5	0.108
**Mesolimbic** (PN; **Figure 6A**)	LS	10.70	0.121
	BSTL	7.56	0.151
	AcbC	7.2	0.083
Total length	-	25.46	0.121
**Mesostriatal** (lateral PBP; **Figure 7A**)	CPu	229.82	0.104
	LSS	5.56	0.103
Total length	-	235.38	0.103

*CE, coefficient of error; For other abbreviations see list of abbreviations*.

##### Mesocortical neurons

**Figure 5:**
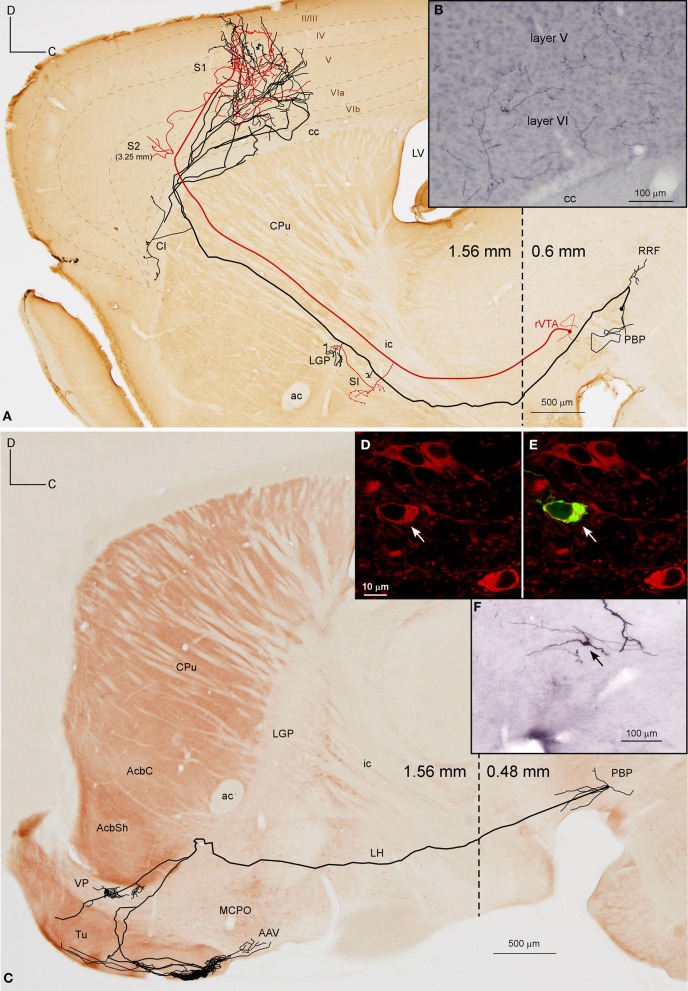
**Mesocortical and mesolimbic neurons. (A)** Sagittal reconstruction of two mesocortical neurons superimposed over a calbindin-stained composition of two different mediolateral levels (0.60 and 1.56 mm separated by a dashed line) according to the atlas of Franklin and Paxinos (2007). The collateral at PBP innervates the contralateral PBP. **(B)** Terminal arborizations at cortical S1 deep layers from the two neurons. **(C)** Sagittal reconstruction of a PBP mesolimbic neuron superimposed over an acetylcholinesterase-stained composition in which the dashed line separates the more medial caudal part (0.48 mm) from the more lateral rostral part (1.56 mm). **(D,E)** Confocal images showing the soma of the neuron depicted in **(C)** filled with viral vector Sindbis-pal-eGFP (green) and its TH immunoreactivity (red). **(F)** Photomicrograph of the somatodendritic domain of the neuron.

Two neurons out of 30 (6.6%) had axons that innervated the S1 cortical area profusely and almost exclusively (Figure 5A, Table 4). Their cell bodies were located in the caudal sector of PBP at 0.6 mm and in the rVTA at 0.48 mm to midline (see **Figures 8D,E,G**). The neurons had ovoid or fusiform perikarya from which emerged 2 to 3 poorly branched primary dendrites.

The axon of the neuron located in PBP (Figure 5A), after emitting a local collateral that innervated the contralateral PBP, ran rostrally, giving two short collaterals that lightly targeted the SI and the lateral globus pallidus (LGP) and then traversed the CPu, emitting only one branch toward Cl. Finally, the main axon entered the S1 cortical area, branching into numerous thin processes scattered in all its layers (Figure 5B). The axon of the second neuron from rVTA was similar to the first except that it also innervated the S2 moderately (Figure 5A). Both reconstructed mesocortical neurons were located in the same hemisphere of the same animal, so both axons merged at S1, making it impossible to separate the terminal fields provided by each neuron. Therefore, the cortical highly dense field at S1 depicted in Figure 5A actually corresponds to two mesocortical neurons.

##### Mesolimbic neurons

A total of 12 neurons out of 30 (40%) had axons that innervated limbic basal forebrain structures such as the Tu, AcbSh, VP, LS, BSTL and amygdala, as well as the AcbC and CPu. They were located at PBP (5 neurons), PN (5 neurons) and rVTA (2 neurons; Table 4) and the DAergic phenotype of two neurons in PN, one in PBP and one in rVTA was confirmed (see **Figures 8B–E,G**). It is noteworthy that all the PN neurons reconstructed in the present study were mesolimbic and that major targets of this neuronal type, namely Acb and Tu, were not innervated by the same neurons. The mesolimbic neurons had ovoid, fusiform or polygonal perikarya with 3–4 poorly branched dendrites.

The Tu, frequently together with VP, received innervation from four neurons located ventrally in VTA. The axon of a DAergic neuron from the ventral aspect of PBP (mediolateral level 0.48 mm) (Figures 5C–F) traveled through LH and SI bifurcating at AcbSh with one branch innervating the VP, and the other entering the Tu sprouting numerous varicose terminal fibers at its most caudoventral sector. The length of the terminal axonal arbor at Tu and VP was 86.82 and 6.14 mm, respectively (Table 5). The other three cells were located at PN: one DAergic neuron also collateralized at VP and arborized profusely in Tu and ICj; another one targeted the deepest Tu layers as well as VP, the superficial layer of Pir and the amygdala, and was the only neuron from this group that provided local collaterals at PN and PBP; the third axon sparsely innervated the Tu as well as other structures (see Table 4 for further details on each neuronal arborization pattern).

**Figure 6:**
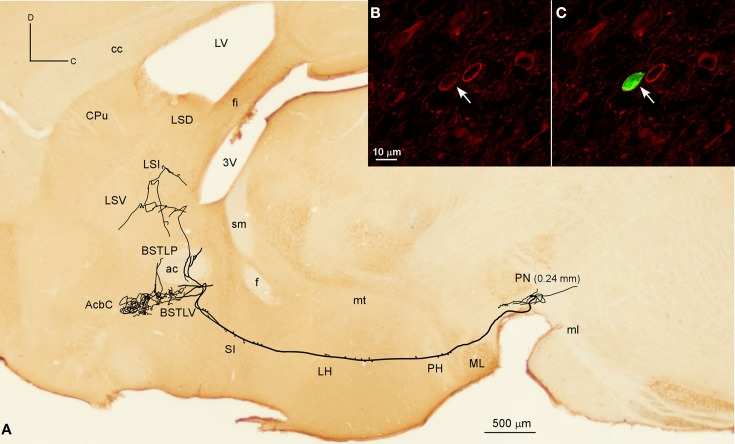
**Mesolimbic neuron. (A)** Sagittal reconstruction of a single mesolimbic neuron located in PN superimposed over a calbindin-stained section corresponding to 0.48 mm lateral from midline (the mediolateral level of the soma is indicated between parentheses). **(B,C)** Confocal images showing the soma filled with Sindbis-pal-eGFP viral vector (green) expressing TH immunoreactivity (red).

The Acb was targeted by another six neurons, whose axons, after traveling through LH and SI without giving any collaterals, provided different arborization patterns. Two of these neurons arose from the lateral aspect of PBP (mediolateral level 0.6 mm) and had the AcbC as their only target, providing a large terminal field, whereas the other four neurons also innervated other structures, mainly the CPu, branching at Acb to a lesser extent. One of them was located at medial PBP (0.36 mm) and moderately innervated the contralateral AcbC and central aspect of the CPu. Another two neurons from rVTA (0.6 mm), one of them DAergic, moderately reached the lateral AcbSh together with the CPu (its dorsocaudal corner in the DAergic case), and its ventral part in the other. Finally, a DAergic PN neuron (Figures 6A–C) gave *boutons en passant* in LH before branching in the ventral part of the lateral division of the BST (BSTLV), AcbC and LS (Table 4). There were two other neurons that exclusively targeted the LS (PN neuron; mediolateral level 0.24 mm) or the ventrolateral sector of CPu and the CeC (PBP neuron; mediolateral level 0.36 mm; Table 4).

##### Mesostriatal neurons

**Figure 7:**
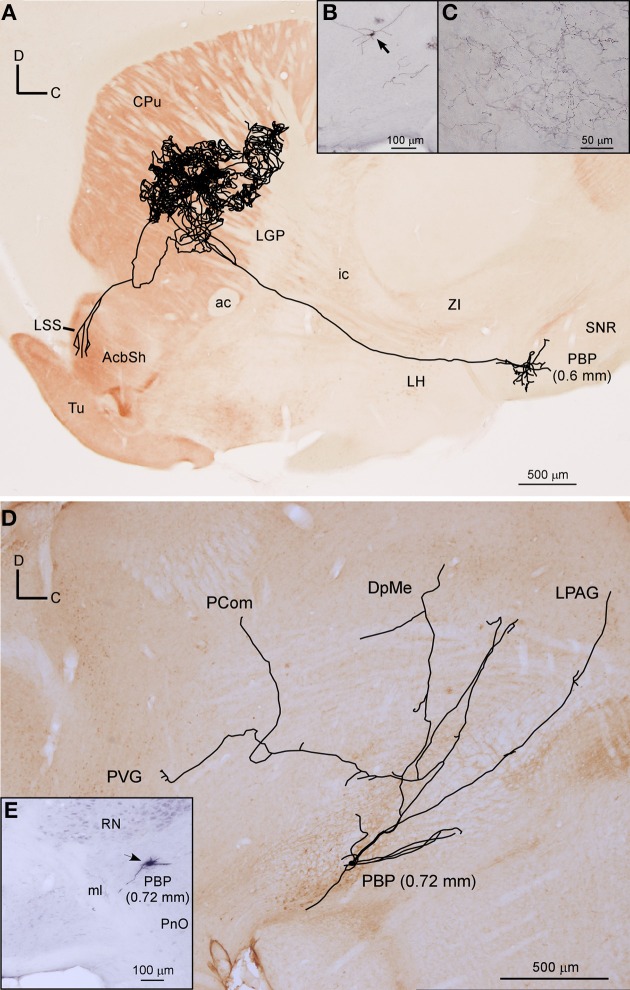
**Mesostriatal and forebrain- and brainstem-projecting neurons (F + BSPN). (A)** Sagittal reconstruction of a single mesostriatal neuron located at PBP superimposed over an acetylcholinesterase-stained section corresponding to 1.56 mm lateral from midline (the mediolateral level of the soma is indicated between parentheses). **(B)** Photomicrograph of the neuron whose axon is shown in **(A)**. **(C)** Terminal fibers at the central aspect of CPu. **(D)** Sagittal reconstruction of a single F + BSPN located at PBP superimposed over a calbindin-stained section corresponding to 0.24 mm (the mediolateral level of the soma is indicated between parentheses). **(E)** Cell body of the neuron whose axon is shown in **(D)**.

**Figure 8:**
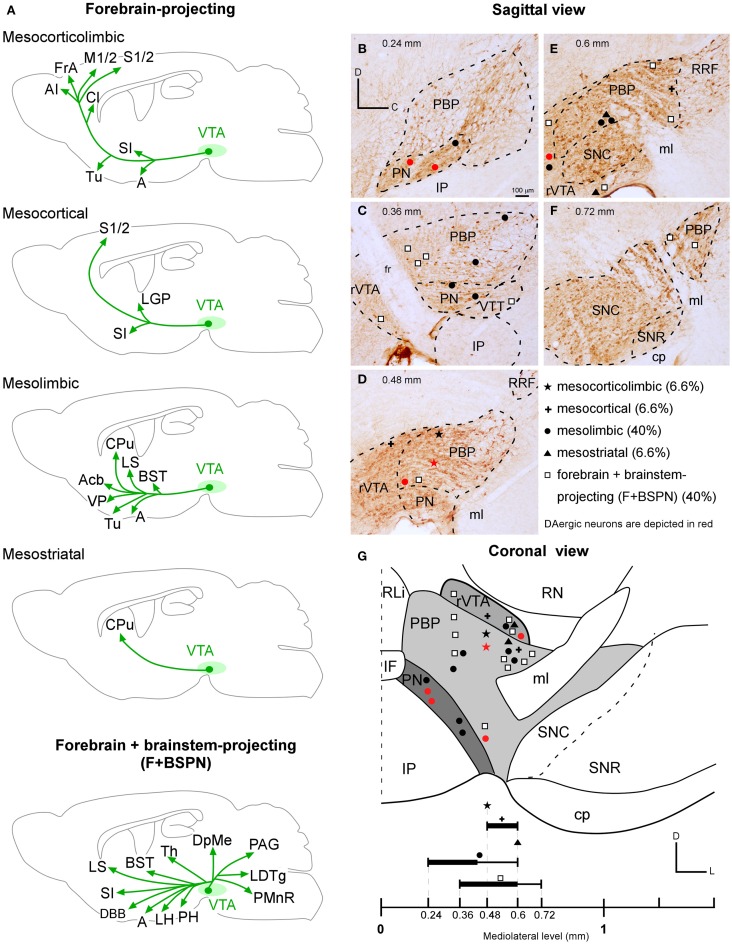
**VTA projection neuron phenotypes. (A)** Drawings of the main structures innervated by the forebrain- (mesocorticolimbic, mesocortical, mesolimbic, mesostriatal) and the forebrain- and brainstem- projecting neuron (F + BSPN) phenotypes. Note that for each neuron type, the drawing represents the main structures innervated by the sum of all the neurons of that type reconstructed in the present study. **(B–G)** Location of the cell bodies of the various neuronal types at PBP, PN, rVTA and VTT, as seen in the sagittal **(B–F**) and coronal (**G**) planes. The relative proportion of each VTA projection neuron phenotype is indicated. The black lines at the bottom are thicker at the levels at which the neurons of a given type are especially abundant.

Two neurons out of 30 (6.6%) had axons that headed directly to the striatum passing through LH and the internal capsule without providing either collaterals or terminal boutons along their paths. The neurons were located at 0.6 mm lateral to the midline in PBP and rVTA (Figures 7A–C, 8E; Table 4), and had ovoid-shaped perikarya from which emerged 3 to 5 frequently branched dendrites. The axon from the PBP neuron branched profusely within the central sector of the CPu, producing a dense terminal arbor with a length of 229.82 mm, and also giving a single collateral at the LSS (Figure 7A). The other neuron targeted the dorsal part of the CPu and the subcallosal stripe providing a heavy terminal field with numerous varicose branches.

#### Forebrain and brainstem-projecting neurons (F + BSPN)

Twelve neurons out of 30 (40%) had axons that branched profusely in proximity to their soma and innervated forebrain and brainstem structures. These neurons had ovoid or fusiform perikarya from which emerged 3 to 5 dendrites. None of these F + BSPN reconstructed in the present study was demonstrably DAergic. In contrast to other highly ramified VTA neurons such as the mesocorticolimbic ones, whose main axons exited the VTA region and could be traced upstream to their final targets, the axons of this type of neuron broke out into numerous collaterals of similar thicknesses within VTA, thus precluding the identification of a putative main axonal branch, and the terminal fields provided by these collaterals at the target structures were very limited. The terminal axons at the target structures provided by these F + BSPN had nevertheless, very similar morphological features to those provided by other DAergic VTA neurons (see Image 1 in Supplementary Material). These neurons were focused on the innervation of brainstem structures but they emitted at least one collateral, typically independent from those innervating the brainstem, that headed rostrally to innervate diencephalic and/or non-cortical prosencephalic targets. An example of this type of neuron is depicted in Figures 7D,E, showing the short dorsocaudal trajectory of the axon through RRF and its early sprouting of weak collaterals at DpMe, LPAG, the nucleus of the posterior commissure (PCom) and the periventricular gray matter (PVG). For a detailed description of each neuronal arborization pattern, see Table 4.

The hypothalamus was frequently innervated by these neurons (9 out of 12) scattered through the four VTA subdivisions; the LH and posterior hypothalamic nucleus (PH) were the main targets of three PBP cells. The nucleus of the horizontal limb of the diagonal band of Broca (HDB) was the most distant rostral target for four cells from PBP and rVTA, LH was reached by one rVTA and one VTT cells and another two PBP neurons innervated the BM, CeM and Me amygdaloid nuclei. Other axons only reached SI and BST or the thalamus, as was the case of a lateral PBP neuron (0.72 mm) that targeted the contralateral mediodorsal thalamic nucleus (MD) after providing sparse collaterals at several contralateral mesencephalic structures as well as a remarkable bilateral innervation at the inferior olivary nucleus. In contrast, the rest of the terminals at the thalamus were seen at medial thalamic nuclei [reuniens (Re), rhomboid (Rh), submedius (Sub), and paraventricular (PV)] and were provided by rVTA or VTT neurons. Regarding brainstem innervation, half of the neurons provided local collaterals, which were sometimes contralateral, at VTA, SNC, and RRF. The periaqueductal gray matter (PAG) and the laterodorsal tegmental nucleus (LDTg) were also common targets of these neurons, and in some cases they were the main structure reached by the corresponding rVTA or VTT cell. Other important caudal targets were the pontine reticulo tegmental nucleus (RtTg) or the dorsal (DR) or the paramedian (PMnR) raphe nuclei.

### Comparison of the length of terminal axonal arbors provided by different VTA neuron types

The amount of innervation that mesocorticolimbic, mesolimbic and mesostriatal VTA neurons provide at their main target sites is depicted in Table 5. The longest length of terminal axonal arbor was found in the dorsal CPu (229.82 mm) innervated by a mesostriatal neuron located at the lateral sector of PBP. Terminal arbors at Tu also tended to be dense, though the amount of fibers was much lower than in CPu, and varied considerably depending on the neuronal type that provided the field. Thus, the length of axonal arbor at Tu provided by a mesolimbic neuron (86.82 mm) was more than double that given by a mesocorticolimbic one (31.92 mm), even though both neurons were located in the same VTA subdivision (medial aspect of PBP), suggesting that the length of terminal arbor that a VTA neuron provides within a given structure varies depending on its pattern of axonal branching, and seems unrelated to the location of the cell body in one VTA subdivision or another. In the mesocorticolimbic neuronal type the terminals at the cerebral cortex were double those at Tu. Terminals at LS, BSTL, and Acb were rather sparse for one neuron that innervated these three targets simultaneously. In terms of the length of the total terminal axonal arbor provided by single neurons the highest value corresponded to the mesostriatal neuron, with 235.38 mm; a length that doubles that of the mesocorticolimbic and mesolimbic neurons. It is also worth noting that the length of the total terminal axonal arbor provided by two mesolimbic neurons was very different, one was 100.5 mm and the other 25.46 mm (Table 5).

## Discussion

The present study was designed to elucidate the cellular diversity of the mouse VTA projection neurons and whether the various cytoarchitectonic subdivisions of VTA innervate different or equivalent sets of targets. To explore these questions, we first examined the brain structures containing terminal axonal fields labeled from small deposits of the anterograde BDA tracer placed in a single VTA subdivision. This analysis demonstrated that small numbers of neurons located in PBP and PN project to widely distributed cortical and subcortical prosencephalic and brainstem structures, indicating either that these VTA subdivisions are populated by distinct and closely intermingled projection neuron types or that their projection neurons have extensively ramified axons. To ascertain whether the patterns of BDA labeling reflected the axonal morphology of individual cells or a mixture of different cell phenotypes, we undertook the task of labeling single neurons located at different VTA subdivisions and tracing their entire axonal arborization. Our findings indicate that there are two main VTA projection neuron phenotypes: (1) neurons whose main axons follow a forward trajectory to innervate cortical and/or basal forebrain structures, and (2) neurons with main axons that branch abundantly in the proximity of the cell body and project toward the forebrain and brainstem. Moreover, the projection targets of the forebrain-projecting neurons distinguished four different types: neurons that project to the neocortex and basal forebrain (mesocorticolimbic), other neurons that almost exclusively innervate the neocortex (mesocortical), others whose axons project to the basal forebrain AcbC and CPu (mesolimbic), and mesostriatal neurons that extensively innervate the CPu. We also used immunohistochemistry against TH to examine the DA phenotype of the neurons whose axons were reconstructed and confirmed that DA was present in the forebrain-projecting neurons.

### Methodological considerations

#### Subdivisions of VTA

In the atlas of Franklin and Paxinos (2007) the VTA is divided into PBP, PN, rVTA and PIF, the latter being a region located ventral to PBP, rostral to PN and caudal to the fr, that extends from 0.24 to 0.48 mm lateral to midline (Franklin and Paxinos, 2007; Fu et al., 2012). The rVTA occupies the rostrodorsal VTA region (Fu et al., 2012), rich in glutamatergic neurons (Yamaguchi et al., 2011; Gorelova et al., 2012). Ikemoto (2007) distinguished four VTA subdivisions: PBP, PN, parafasciculus retroflexus area (PFR), and VTT. The PFR would encompass rVTA and the rostral PIF, and be a DAergic cell-body-poor zone continuing with TH^+^ cell bodies in the hypothalamic area. The VTT is located just posterior to the PN and lateral to the posterior half of the IP, and the density of TH^+^ cell bodies is clearly lower than in PN and PBP. This region as outlined by Ikemoto (2007) does not represent what is currently identified as the tVTA/RMTg, a major GABA brake for DA systems (Jhou et al., 2009; Barrot et al., 2012; Bourdy and Barrot, 2012; Bourdy et al., 2014; Yetnikoff et al., 2014). According to some authors (Swanson, 1982; Halliday and Törk, 1986; Ikemoto, 2007; Yamaguchi et al., 2011), the midline nuclei IF, RLi, and CLi do not belong to the VTA, whereas other investigators have included these nuclei with the A10 group, even though they contain DAergic neurons with chemical features that are distinct from those located more laterally (Del-Fava et al., 2007; Fu et al., 2012).

#### Visualization of the DAergic phenotype of the Sindbis-pal-eGFP infected neurons

The Sindbis-pal-eGFP vector used in the present study was produced and donated by Dr. Takahiro Furuta and Dr. Takeshi Kaneko (Kyoto University, Japan) (see Furuta et al., 2001 for details about the construction of recombinant Sindbis viruses). This vector is highly efficient for visualizing the whole arborization of single axons in their entirety because of the massive synthesis of GFP protein once a codifying RNA, in principle a single one, has entered a cell (Kuramoto et al., 2009; Matsuda et al., 2009). Next, we performed immunofluorescence staining for TH to determine the DAergic phenotype of the infected neurons. In our study, of the 30 GFP-labeled VTA neurons located among many TH^+^ cell bodies, only five were demonstrated to express TH under confocal microscopy. We consider that this low quantity of infected TH^+^ neurons is quite striking since 62% of VTA neurons are DAergic (Nair-Roberts et al., 2008), and the infected neurons were scattered among abundant TH^+^ cells. The suspicion that something could be hampering the expression of TH in many Sindbis-pal-eGFP infected VTA neurons was strongly supported when we performed a large injection of the fluorescent tetramethylrodamine anterograde tracer in VTA, and found that of the 24 neurons that were labeled by the tracer, 12 expressed TH immunostaining. One possible explanation for the low rate of Sindbis-pal-eGFP infected neurons being TH^+^ could be related to an overproduction of GFP protein driven by the strong subgenomic promoter of the Sindbis vector, which would massively consume energy and nutrient resources in the infected neurons, shutting off the synthesis of proteins such as TH by the infected host cell (Jeromin et al., 2003). We consequently suspect that many of the reconstructed TH-negative neurons might have actually been DAergic. The half-life of the TH protein is 30 h (Tank et al., 1986), so an infected cell whose TH content could be visualized by immunofluorescence might correspond to a neuron that still had enough enzyme, maybe because it had been infected later due to the ability of the viral vector to stay in the extracellular space an undetermined period of time before entering a neuron. Another possible explanation for the low rate of Sindbis-pal-eGFP infected neurons being TH^+^ could be that the vector has some kind of incompatibility with DAergic cells, thus infecting preferentially non-DAergic neurons.

It should be noted that in the study of the nigrostriatal pathway by Matsuda et al. (2009) only 21 out of 70 (30%) nigral neurons infected with the same Sindbis viral vector were demonstrated to be positive for TH immunoreactivity—a surprisingly low rate for a structure that contains mostly DAergic neurons. This finding is still more striking when the survival time used in that study was even shorter than the one used here, ranging from 36 to 42 h.

### Target structures of VTA neurons

The present study has analyzed the anterograde labeling from BDA deposits located in PBP and PN, and reconstructed the axons of neurons located in PBP, PN and rVTA. In this section we will review the projections of these VTA subdivisions in mice.

#### Cerebral cortex

Numerous studies have reported an extensive cortical innervation with origin in VTA (Beckstead et al., 1979; Swanson, 1982; Oades and Halliday, 1987; Carr and Sesack, 2000; Hosp et al., 2011, 2015). In the present study we have visualized wide cortical innervation from PBP neurons, whose axons targeted sensory (somatosensory), motor, limbic (retrosplenial, cingular, entorhinal), prefrontal (prelimbic), and association (frontal, orbital) territories. Within the motor and somatosensory cortices, the axon terminals labeled from PBP injections innervated primary and secondary territories. This finding in rodents resembles descriptions in cats and primates in which the ventral mesencephalon targeted motor areas 4 and 6 (Reinoso Suárez and Llamas, 1968; Williams and Goldman-Rakic, 1998). According to the results shown here, the projections from PBP to the M1 or S1 cortices could have origin in neurons that are different from those that target M2 or S2 territories; though larger number of such neurons needs to be traced in the future so as to confirm these preliminary observations. Furthermore, one PBP neuron that targeted primary cortices was demonstrably DAergic (Table 4). This finding indicates that PBP holds neurons focused on exclusively innervating either primary or secondary cortices, and that at least some of those targeting M1 and S1 are DAergic. The selectivity that some PBP neurons show toward innervating primary or secondary sensorimotor territories strengthens the functional specificity of the DA innervation of M1 and M2, which in M1 is necessary for motor skill learning (Hosp et al., 2011), and in M2 for the evaluation and selection of voluntary action driven by the expected reward and reward prediction error conveyed by DAergic midbrain neurons (Schultz, 1998; Sul et al., 2011).

Our material only showed terminals in the PrL territory of the prefrontal cortex from injections located at PBP, but PN neurons are also known to provide DA inputs to the medial prefrontal cortex in rodents and primates (Albanese and Minciacchi, 1983; Deutch et al., 1991; Williams and Goldman-Rakic, 1998; Carr and Sesack, 2000; Lammel et al., 2008). The entorhinal cortex receives input from both PBP and PN, though the intensity of innervation provided by the latter is notably higher than that from PBP, suggesting a target preference as previously suggested (Swanson, 1982; Scheibner and Törk, 1987). According to our results, PBP is likely to project to the cerebral cortex more abundantly than does PN. This is based on the large quantity of cortical territories innervated from BDA deposits at PBP in comparison with those labeled from PN, and also on the fact that of the nine PBP neurons whose axon followed a forward route three provided cortical input, whereas of the five reconstructed PN axons following a forward route none projected to the cerebral cortex.

#### Amygdala, hippocampus, septal nuclei and diagonal band of Broca

The extended amygdala (Ce, Me and BST) is another well-documented target of VTA neurons (Ungerstedt, 1971; Beckstead et al., 1979; Swanson, 1982; Oades and Halliday, 1987; Laviolette and Grace, 2006; Present study). This projection is mostly DAergic, although GABAergic afferents have also been recently reported (Taylor et al., 2014). We provide new information regarding the selectivity of various VTA subdivisions in innervating specific amygdaloid nuclei. Thus, the PBP projects to the Ce, Me and BL, whereas the PN neurons do not, instead targeting the Co. Other regions such as the AAV and the BST are innervated by PBP and PN neurons. The DAergic nature of the projection from PBP to the BLA, Ce and Me, and from PN to BST has been shown here, but the possibility that these projections also express VGluT2 should not be discarded, at least in mice (Taylor et al., 2014). BST DAergic terminals participate in reward prediction and mechanisms for drug abuse (Eiler et al., 2003; Krawczyk et al., 2011), while noradrenergic terminals are implicated in withdrawal symptoms through the inhibition of BST back projections to VTA that exert a potent disinhibitory control over DAergic neurons (Krawczyk et al., 2011; Kudo et al., 2014). All the VTA subdivisions analyzed here innervated the LS, and those from PN provide DA to this region, which mediates the response to both rewarding and stressing stimuli (Sotomayor et al., 2005) and is part of a circuit connecting the dorsal hippocampus and VTA related to context-reward associations (Luo et al., 2011). The diagonal band was targeted by PBP, PN and rVTA, whereas the hippocampus only received terminals from PN neurons, which targeted the subiculum. In this study we failed to detect projections from VTA to other known targeted hippocampal structures such as the stratum oriens of CA1 and CA3 (see Yetnikoff et al., 2014 for references).

#### Striatum and pallidum

Our study reveals that PBP and PN contribute to innervating the Tu, AcbSh, AcbC and VP, structures that in turn project back to VTA and are important for reward-related behaviors (Zahm et al., 2001; Geisler and Zahm, 2005; Geisler et al., 2007; Ikemoto, 2007; Tripathi et al., 2010, 2013). The neurons that innervate Acb are abundant in PN, which provides DA to the AcbC, as well as the entire mediolateral extent of PBP, and these neurons are different from those that target VP and Tu, which predominate in PN and in the medial PBP. The phenotype of the VTA neurons that innervate these basal forebrain structures is probably different, because while the Acb receives more DA than GABA and glutamate from VTA, the opposite pattern is observed in VP and Tu (Hnasko et al., 2012; Taylor et al., 2014). Though VTA is targeted by many VP neurons (Tripathi et al., 2013), direct projections from VTA to VP are likely to be scarce (only 4 out of 30 neurons targeted the VP) and composed of axon collaterals, some of which may be DAergic, and which headed to the Tu-ICj, providing approximately six times more axon length in Tu than in VP. As was expected in the light of previous studies reporting the continuity between the dorsal tier of SNC and lateral PBP (Haber and Fudge, 1997), the dorsal CPu was the only target of VTA neurons laterally located in PBP and rVTA, and the axons provided terminal fields containing a greater axon length than those located in other VTA targets.

#### Thalamus and epithalamus

Projections from VTA to the LHb and the medial thalamus are well known (Beckstead et al., 1979; Swanson, 1982; Albanese and Minciacchi, 1983; Skagerberg et al., 1984; Oades and Halliday, 1987; Domesick, 1988) and contribute to reward regulation. In the case of LHb, VTA terminals can release GABA to inhibit LHb neurons and promote reward (Stamatakis et al., 2013). Other VTA terminals at LHb release glutamate (Hnasko et al., 2012), which may serve to indirectly inhibit VTA DAergic neurons through activation of inhibitory neurons in the rostromedial tegmental nucleus (Hong et al., 2011). The important network between VTA and LHb deserves further investigation at the single neuron level since none of the reconstructed neurons in the present study projected to LHb. The PBP, PN, and rVTA targeted the thalamus, although they provide only scarce labeling concentrated on the anterior (AM), midline (PV, CM, Rh, Re, Sub), MD and reticular (Rt) nuclei, without a clear preference by any VTA subdivision toward innervating specific thalamic nuclei.

#### Hypothalamus and periaqueductal gray matter

The anterior, lateral, and posterior hypothalamic areas, the supraoptic area and the medial mammillary nucleus receive direct projections from VTA neurons. The LH receives dense DAergic and GABAergic VTA inputs (Taylor et al., 2014), and in turn it innervates VTA DAergic neurons involved in reward-value coding (Watabe-Uchida et al., 2012). This hypothalamic region is a common target of the VTA subdivisions analyzed here, and PN neurons are one of its DA sources. These VTA regions also innervate the PAG, mostly concentrated on its lateral aspect, and at least some PBP neurons provide DA to this region. The PAG innervation was specially abundant and bilateral from VTT, which contains many PAG-projecting GABA neurons related with pain and stress regulation (Kirouac et al., 2004).

#### Brainstem nuclei

Interactions among VTA neurons may be mediated by varicose dendrites and local, topographically organized axonal connections (Adell and Artigas, 2004; Ferreira et al., 2008). Intra VTA axonal terminals abounded after injecting PBP neurons, which provided innervation to the PBP itself and less frequently to PN, IF, IP, and rVTA. Less frequent local innervation was found after injecting PN and terminals were observed in PN, PBP, CLi, and rVTA. As observed in rats (Ferreira et al., 2008), projections to SNC and RRF arose exclusively from PBP neurons. Labeled fibers descending from PBP and PN innervate the dorsal raphe nucleus, pedunculotegmental nucleus (PTg) and LDTg, as documented in earlier anatomical studies (Beckstead et al., 1979; Cornwall et al., 1990), and also the inferior olivary nucleus, which was innervated by a PBP neuron in our study, and from the RLi in another previous study (Del-Fava et al., 2007).

### VTA neuron projection phenotypes

Our findings indicate that VTA contains two main types of projection neurons: forebrain-projecting neurons (60%), and F + BSPN (40%). We have also shown that on the basis of projection targets the forebrain-projecting neurons include: cells that project to the cerebral cortex and basal forebrain (mesocorticolimbic; 6.6%), other neurons whose axons almost exclusively innervate the cerebral cortex (mesocortical; 6.6%), or the basal forebrain, AcbC and CPu (mesolimbic; 40%), and mesostriatal neurons that extensively innervate the CPu (6.6%; Figure 8). These findings demonstrate for the first time at the single cell level that there are segregated mesocortical and mesolimbic output systems, made of axons of each of these types of neurons, as well as a distinct mesocorticolimbic output system composed of axons from VTA neurons that simultaneously innervate the cerebral cortex and subcortical limbic structures.

In agreement with previous investigations (Berger et al., 1976; Lindvall et al., 1978; Simon et al., 1979; Fallon, 1981; Swanson, 1982; Albanese and Minciacchi, 1983; Tzschentke, 2001), we have observed that the VTA neurons that target the cerebral cortex (i.e., the mesocortical and mesocorticolimbic) arise mostly from PBP, while PN harbors neurons whose axons innervate subcortical limbic territories. Nevertheless, it is important to note that none of the neuronal types described in this study on the basis of their axonal branching patterns is distributed in a manner that correlates with the four VTA subdivisions analyzed here (Figures 8B–G). The topographic arrangement of the various VTA cell types analyzed here suggests that the mesolimbic, mesocorticolimbic, mesocortical, and mesostriatal VTA neurons are distributed following a mediolateral sequence, whereas the F + BSPN seem to be widely scattered along the mediolateral extent of VTA (Figure 8G). The mesolimbic neurons were abundant in PBP, PN and rVTA, with 66% of them being concentrated from the midline to 0.48 mm. The mesocorticolimbic neurons were confined to the medial sector of PBP (0.48 mm; Figures 8D,G), a region that contains many mesoprefrontal cells (Lammel et al., 2008). Mesocortical and mesostriatal neurons were placed in rVTA and PBP, the former from 0.48 to 0.6 mm, and the latter at 0.6 mm. The F + BSPN were spread from 0.36 to 0.72 mm lateral to midline, with 50% of them located medially to 0.6 mm within PBP, rVTA, and the VTT region according to Ikemoto (2007), and the remaining ones in the lateral sector of PBP and rVTA.

#### The forebrain-projecting VTA neurons

Our study describes for the first time the existence of VTA neurons, some of which are DAergic, with axons that collateralize to reach wide territories of the cortex and basal forebrain. This indicates that large number of cortical and basal forebrain structures can respond to the DA signal emitted by a single VTA neuron, at least in mice. Typically, the main axons of these mesocorticolimbic neurons avoid the CPu by passing around it ventrally, then innervating the Cl twice, and going on to target the association, motor, somatosensory, and cingulate cortical territories. These axons also target olfactory structures, the amygdala and VP among others, though the length of the terminal axonal arbor provided at the cortex doubles that at Tu and is almost six times longer than the length at AStr. Because of their widespread collateralization to forebrain targets, the mesocorticolimbic neurons probably act as an integrative node for cortical and limbic territories that are targeted by segregated mesocortical and mesolimbic projections. Of particular interest is the fact that the same DAergic mesocorticolimbic cell reached both the prefrontal cortex and the BLA, since connections between VTA and these two structures have been implicated in the pathogenic mechanisms of schizophrenia in humans (Laviolette, 2007).

The mesocortical neurons innervated the cerebral cortex in a more focused manner than the mesocorticolimbic neurons, and their terminals were restricted to the somatosensory territory. Another distinctive feature of both types of neurons is that the trajectory of their main axon traversed the CPu diagonally leaving a collateral at LGP. None of the neurons that targeted the cerebral cortex innervated the Acb, supporting the idea of the existence of functionally distinct mesocortical and mesoaccumbal systems (Sesack and Carr, 2002; Björklund and Dunnett, 2007). The VTA neurons that innervated the Acb, either the core or the shell, showed a predominant innervation of this structure, being the only target of some of the neurons (Table 4), suggesting a powerful and selective control of VTA over the ventral striatopallidal system that could parallel the control exerted by the SNC over the CPu. However, differently from the nigrostriatal pathway, which is mostly DAergic, Acb-projection VTA neurons can also release GABA or glutamate (Yamaguchi et al., 2011; Taylor et al., 2014), as well as being able to co-release DA with glutamate (Stuber et al., 2010).

#### The forebrain- and brainstem-projecting neuron type (F + BSPN)

Of the 30 neurons that we reconstructed, 40% were of this type. These neurons occur at rostral and caudal aspects of the VTA and their axons branch abundantly in the proximity of the soma to subtly innervate numerous and diverse structures, but avoid those basal forebrain and cortical targets that typically distinguish DAergic VTA neurons. None of the 12 F + BSPN reconstructed in the present study was demonstrably DAergic. The unusual arborization patterns of this neuron type resemble the reticular formation, and this link is strengthened by the fact that these neurons innervate structures closely associated to it. For instance, the majority of these cells innervated the PH or the LH-preoptic continuum, which are regions that, except for a few *boutons en passage* from some mesolimbic PN axons, do not receive innervation from any other VTA neuronal type. Likewise, the HDB, zona incerta (ZI), and midline thalamic nuclei were exclusively innervated by this type of cell. Regarding brainstem structures, the LPAG and LDTg were the most frequently targeted, the latter structure being a controller of DAergic VTA neuron activity (Lodge and Grace, 2006) and the main driver of DAergic PBP neurons that innervate the lateral AcbSh and subserve reward function (Lammel et al., 2012). Interestingly, the axons of two PBP F + BSPN collateralized at both LDTg and PBP, suggesting an intrinsic VTA regulatory role over the DAergic mesoaccumbens pathway.

### Concluding remarks

The VTA neurons have received great attention because they are the primary source of DA in target structures such as the medial prefrontal cortex and Acb, with important roles in a broad range of motivated behaviors and neuropsychiatric disorders (Björklund and Dunnett, 2007; Ikemoto, 2007; Laviolette, 2007; Lau et al., 2013). In recent years, the chemical heterogeneity of the VTA neurons has become more evident, since a large population of neurons containing GABA and glutamate are intermingled with DAergic neurons in the various VTA subdivisions (Kawano et al., 2006; Nair-Roberts et al., 2008; Morales and Root, 2014). The number and combinations of target structures innervated by single DAergic and non-DAergic VTA neurons had not been examined to date, and consequently the existence of neurons with such diverse and specific anatomical axonal arborizations that might funnel information simultaneously to multiple cortical and subcortical structures, such as the mesocorticolimbic neurons described here, had been unsuspected until now. These findings clearly contrast with the idea that axonal projections originating from VTA neurons are mainly unbranched (see Yetnikoff et al., 2014), although the VTA projection neuron phenotypes that have been described here in mice might differ from the ones existing in other rodents. Our study has also demonstrated the existence of distinctive mesocortical, mesolimbic, and mesostriatal pathways at the single cell level (Björklund and Dunnett, 2007), that are not spatially segregated in VTA. Furthermore, each of these systems encompasses a large diversity of neurons in terms of axonal projection patterns, and very probably also in terms of neurotransmitter content, knowledge of which is needed to gain deeper insight into the anatomical substrate that allows these neurons to participate in specific functional roles (Li et al., 2013; Lammel et al., 2014; Overton et al., 2014; Yetnikoff et al., 2014). The combination of information about the neurotransmitter content, axonal branching patterns and the length of terminal axonal arbor at the targeted areas is an important step toward providing more complete and functionally relevant information on neuron diversity, as well as reflecting the usefulness of the new powerful viral vectors that assure a complete filling of a whole axon.

## Author contributions

AA: acquisition of the data related to microdeposits and single neurons. Analysis, interpretation and drafting the results. CR: acquisition of the data related to microdeposits and single neurons. Analysis, interpretation and drafting the results. AA and CR contributed equally to this study. MG: acquisition of the data related to length of terminal axonal arbors. Analysis, interpretation and drafting the results. FC: substantial contributions to the design of the work. Interpretation of data, and revising the manuscript for important intellectual content. LP: design of the work. Analysis, interpretation of data and writing of the whole manuscript.

### Conflict of interest statement

The authors declare that the research was conducted in the absence of any commercial or financial relationships that could be construed as a potential conflict of interest.

## Abbreviations

A: amygdaloid complex
AAV: anterior amygdaloid area, ventral part
Acb: accumbens nucleus
AcbC: accumbens nucleus, core
AcbSh: accumbens nucleus, shell
ACo: anterior cortical amygdaloid area
AH: anterior hypothalamic area
AHA: anterior hypothalamic area, anterior part
AHC: anterior hypothalamic area, central part
AHP: anterior hypothalamic area, posterior part
AI: agranular insular cortex
AID: agranular insular cortex, dorsal part
AIV: agranular insular cortex, ventral part
AM: anteromedial thalamic nucleus
AO: anterior olfactory area
AOM: anterior olfactory area, medial part
AOP: anterior olfactory area, posterior part
AOV: anterior olfactory area, ventral part
APT: anterior pretectal nucleus
AStr: amygdalostriatal transition area
B: basal nucleus (Meynert)
BL: basolateral amygdaloid nucleus
BLA: basolateral amygdaloid nucleus, anterior part
BM: basomedial amygdaloid nucleus
BMP: basomedial amygdaloid nucleus, posterior part
MPA: medial preoptic area
BST: bed nucleus of the stria terminalis
BSTL: bed nucleus of the stria terminalis, lateral division
BSTLI: bed nucleus of the stria terminalis, lateral division, intermediate part
BSTLD: bed nucleus of the stria terminalis, lateral division, dorsal part
BSTLP: bed nucleus of the stria terminalis, lateral division, posterior part
BSTLV: bed nucleus of the stria terminalis, lateral division, ventral part
BSTMA: bed nucleus of the stria terminalis, medial division, anterior part
BSTMPI: bed nucleus of the stria terminalis, medial division, posterointermediate part
BSTMPL: bed nucleus of the stria terminalis, medial division, posterolateral part
Ce: central amygdaloid nucleus
CeC: central amygdaloid nucleus, capsular part
CeL: central amygdaloid nucleus, lateral division
CeM: central amygdaloid nucleus, medial division
Cg: cingular cortex
Cg1: cingular cortex, area 1
Cg2: cingular cortex, area 2
Cl: claustrum
CLi: caudal linear nucleus
CM: central medial thalamic nucleus
Co: cortical amygdaloid nucleus
CPu: caudate-putamen
DBB: diagonal band of Broca
DEn: dorsal endopiriform claustrum
Dk: nucleus of Darkschewitsch
DMPAG: dorsomedial periaqueductal gray
DP: dorsal peduncular cortex
DpMe: deep mesencephalic nucleus
DpG: deep gray layer of the superior colliculus
DR: dorsal raphe nucleus
DRD: dorsal raphe nucleus, dorsal part
DRV: dorsal raphe nucleus, ventral part
DTT: dorsal tenia tecta
DTg: dorsal tegmental nucleus
DTgP: dorsal tegmental nucleus, pericentral part
E/OV: ependymal and subendymal layer/olfactory ventricle
FrA: frontal association cortex
F + BSPN: forebrain- and brainstem-projecting neurons
GI: granular insular cortex
HDB: nucleus of the horizontal limb of the diagonal band
ICj: island of Calleja
IF: interfascicular nucleus
IO: inferior olivary nucleus
IPAC: interstitial nucleus of the posterior limb of the anterior commissure
IP: interpeduncular nucleus
LEnt: lateral entorhinal cortex
LDTg: laterodorsal tegmental nucleus
LGP: lateral globus pallidus
LH: lateral hypothalamic area
LHb: lateral habenular nucleus
LHbM: lateral habenular nucleus, medial part
LO: lateral orbital cortex
LPAG: lateral periaqueductal gray
LPO: lateral preoptic area
LS: lateral septal nucleus
LSD: lateral septal nucleus, dorsal part
LSI: lateral septal nucleus, intermediate part
LSS: lateral stripe of the striatum
LSV: lateral septal nucleus, ventral part
M1: primary motor cortex
M2: secondary motor cortex
MCLH: magnocellular nucleus of the lateral hypothalamus
MCPO: magnocellular preoptic nucleus
MD: mediodorsal thalamic nucleus
MDL: mediodorsal thalamic nucleus, lateral part
MDM: mediodorsal thalamic nucleus, medial part
Me: medial amygdaloid nucleus
MeAD: medial amygdaloid nucleus, anterodorsal part
MEnt: medial entorhinal cortex
MHb: medial habenular nucleus
ML: medial mammillary nucleus, lateral part
MM: medial mammillary nucleus, medial part
MO: medial orbital cortex
MPT: medial pretectal nucleus
MS: medial septal nucleus
OT: nucleus of the optic tract
PAG: periaqueductal gray matter
PBP: parabrachial pigmented nucleus of the VTA
PCom: nucleus of the posterior commissure
PFR: parafasciculus retroflexus area
PH: posterior hypothalamic nucleus
Pir: piriform cortex
PMCo: posteromedial cortical amygdaloid area
PMnR: paramedian raphe nucleus
PN: paranigral nucleus
Pn: pontine nuclei
PnO: pontine reticular nucleus, oral part
PnV: pontine reticular nucleus, ventral part
PPT: posterior pretectal nucleus
PRh: perirhinal cortex
PrL: prelimbic cortex
PSTh: parasubthalamic nucleus
PTg: pedunculotegmental nucleus
PV: paraventricular thalamic nucleus
PVA: paraventricular thalamic nucleus, anterior part
PVG: periventricular gray matter
PVP: paraventricular thalamic nucleus, posterior part
Re: reuniens thalamic nucleus
Rh: rhomboid thalamic nucleus
RI: rostral interstitial nucleus
RM: retromammilar nucleus
Rt: reticular nucleus (prethalamus)
RtTg: reticulotegmental nucleus of the pons
RSA: retroesplenial agranular cortex
RSG: retroesplenial granular cortex
RRF: retrorubral field
rVTA: ventral tegmental area, rostral part
S1: primary somatosensory cortex
S2: secondary somatosensory cortex
SI: substantia innominata
SNC: substantia nigra, compact part
SNR: substantia nigra, reticular part
STh: subthalamic nucleus
Sub: submedius thalamic nucleus
SuMM: supramammillary nucleus, medial part
TH: tyrosine hydroxylase
Tu: olfactory tubercle
VDB: nucleus of the vertical limb of the diagonal band
VEn: ventral endopiriform claustrum
VLPAG: ventrolateral periaqueductal gray
VO: ventral orbital cortex
VP: ventral pallidum
VTA: ventral tegmental area
VTT: ventral tegmental tail
VTt: ventral tenia tecta
ZI: zona incerta.
