# Supplementary figures and images for: Long-range projection neurons of the mouse ventral tegmental area: a single-cell axon tracing analysis
Source: Front Neuroanat. 2015 May 19;9:59. doi: 10.3389/fnana.2015.00059 (PMC4436899; doi:10.3389/fnana.2015.00059)

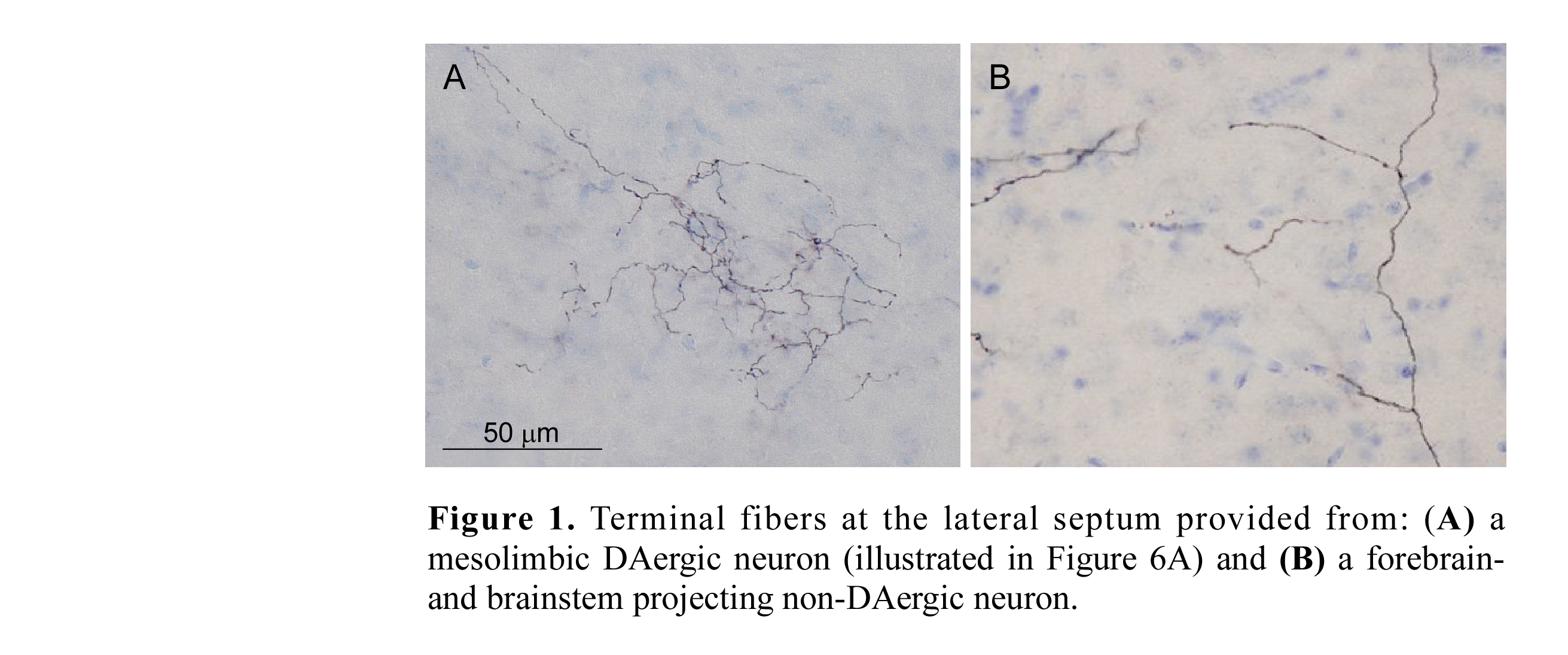

Supplement: Supplementary file 1 [file Image1.TIF]
