# Supplementary material for: Long-range projection neurons of the mouse ventral tegmental area: a single-cell axon tracing analysis
Source: Front Neuroanat. 2015 May 19;9:59. doi: 10.3389/fnana.2015.00059 (PMC4436899; doi:10.3389/fnana.2015.00059)
Supplement: Supplementary file 2 [file Table1.PDF]

Table 1. BDA deposits analyzed in the present study

Mice	Hemisphere	Location of the BDA deposit
**1**	Right	PBP (1*)
	Left	PBP (2*)
**2**	Right	PBP (4*)
	Left	PBP (3*)
**3**	Right	PBP (5*)
	left	Medial lemniscus
**4**	Right	PN (6*)
	Left	-
**5**	Right	PN (7*)
	Left	Medial lemniscus/SN+

***** The number indicates the BDA deposit illustrated in Figure 1.
- The BDA deposit was very weak and did not produce anterograde labeling.
+ The labeling of this BDA deposit was confined mostly to the thalamus and striatum of the ipsilateral hemisphere. Thought the projections of the medial lemniscus and the substantia nigra (SN) are typically ipsilateral, the possibility that some fibers labeled from this deposit could have traversed the midline and contributed to the labeling observed in the contralateral thalamus cannot be ruled out.
