# Supplementary material for: Long-range projection neurons of the mouse ventral tegmental area: a single-cell axon tracing analysis
Source: Front Neuroanat. 2015 May 19;9:59. doi: 10.3389/fnana.2015.00059 (PMC4436899; doi:10.3389/fnana.2015.00059)
Supplement: Supplementary file 3 [file Table2.PDF]

**Table 2. Stereological parameters for estimations of the length of terminal axonal arbors labeled from single neurons**

| Terminal arbor location | ssf µm | tm µm | h (box) µm | a (box) $\mu m^2$ | d (plane) µm | Step length (x and y) µm x µm |
|---|---|---|---|---|---|---|
| [1]CPu | 2 | 17.60 | 10 | 2377 | 20 | 200 |
| [1]LSS | 1 | 21.62 | 10 | 4723 | 10 | 72.5 |
| [2]Tu | 2 | 13.53 | 8 | 1764 | 20 | 100 |
| [2]VP | 1 | 12.48 | 8 | 2343 | 10 | 60 |
| [2]AAV | 2 | 13.41 | 10 | 4723 | 10 | 108 |
| [3]Cerebral cortex | 4 | 18.84 | 10 | 2377 | 10 | 100 |
| [3]Tu | 2 | 20.39 | 10 | 2377 | 10 | 100 |
| [3]AStr | 1 | 20.66 | 10 | 2377 | 10 | 100 |
| [4]LS | 1 | 14.99 | 10 | 2351 | 10 | 100 |
| [4]BSTL | 2 | 17.85 | 10 | 2351 | 10 | 60 |
| [4]AcbC | 1 | 16.64 | 10 | 2351 | 10 | 60 |

[1] The soma of the mesostriatal neuron that provided this terminal field was located in the lateral PBP (Fig. 7A).
[2] The soma of the mesolimbic neuron that provided this terminal field was located in the medial PBP (Fig. 5C).
[3] The soma of the mesocorticolimbic neuron that provided this terminal field was located in the medial PBP (Fig. 4D).
[4] The soma of the mesolimbic neuron that provided this terminal field was located in PN (Fig. 6A).

**Abbreviations:** a (box): sampling box area; AcbC: accumbens nucleus, core; AStr: amydalostriatal transition area; BSTL: bed nucleus of the stria terminalis, lateral division; CPu: caudate-putamen; d (plane): plane separation distance; h (box): height of the sampling box; LS: lateral septal nucleus; ssf: section sampling fraction; tm: mean section thickness; Tu: olfactory tubercle; VP: ventral pallidum.
